# Monodisperse Core-Shell NaYF_4_:Yb^3+^/Er^3+^@NaYF_4_:Nd^3+^-PEG-GGGRGDSGGGY-NH_2_ Nanoparticles Excitable at 808 and 980 nm: Design, Surface Engineering, and Application in Life Sciences

**DOI:** 10.3389/fchem.2020.00497

**Published:** 2020-06-12

**Authors:** Uliana Kostiv, Hana Engstová, Bartosz Krajnik, Miroslav Šlouf, Vladimír Proks, Artur Podhorodecki, Petr Ježek, Daniel Horák

**Affiliations:** ^1^Institute of Macromolecular Chemistry of the Czech Academy of Sciences, Prague, Czechia; ^2^Institute of Physiology of the Czech Academy of Sciences, Prague, Czechia; ^3^Department of Experimental Physics, Wroclaw University of Science and Technology, Wroclaw, Poland

**Keywords:** upconversion nanoparticles, core-shell, 808 nm excitation, luminescence, PEG-neridronate, RGDS peptide, Hep-G2 and HeLa cells

## Abstract

Lanthanide-doped upconversion nanoparticles (UCNPs) have a unique capability of upconverting near-infrared (NIR) excitation into ultraviolet, visible, and NIR emission. Conventional UCNPs composed of NaYF_4_:Yb^3+^/Er^3+^(Tm^3+^) are excited by NIR light at 980 nm, where undesirable absorption by water can cause overheating or damage of living tissues and reduce nanoparticle luminescence. Incorporation of Nd^3+^ ions into the UCNP lattice shifts the excitation wavelength to 808 nm, where absorption of water is minimal. Herein, core-shell NaYF_4_:Yb^3+^/Er^3+^@NaYF_4_:Nd^3+^ nanoparticles, which are doubly doped by sensitizers (Yb^3+^ and Nd^3+^) and an activator (Er^3+^) in the host NaYF_4_ matrix, were synthesized by high-temperature coprecipitation of lanthanide chlorides in the presence of oleic acid as a stabilizer. Uniform core (24 nm) and core-shell particles with tunable shell thickness (~0.5–4 nm) were thoroughly characterized by transmission electron microscopy (TEM), energy-dispersive analysis, selected area electron diffraction, and photoluminescence emission spectra at 808 and 980 nm excitation. To ensure dispersibility of the particles in biologically relevant media, they were coated by in-house synthesized poly(ethylene glycol) (PEG)-neridronate terminated with an alkyne (Alk). The stability of the NaYF_4_:Yb^3+^/Er^3+^@NaYF_4_:Nd^3+^-PEG-Alk nanoparticles in water or 0.01 M PBS and the presence of PEG on the surface were determined by dynamic light scattering, ζ-potential measurements, thermogravimetric analysis, and FTIR spectroscopy. Finally, the adhesive azidopentanoyl-modified GGGRGDSGGGY-NH_2_ (RGDS) peptide was immobilized on the NaYF_4_:Yb^3+^/Er^3+^@NaYF_4_:Nd^3+^-PEG-Alk particles via Cu(I)-catalyzed azide-alkyne cycloaddition. The toxicity of the unmodified core-shell NaYF_4_:Yb^3+^/Er^3+^@NaYF_4_:Nd^3+^, NaYF_4_:Yb^3+^/Er^3+^@NaYF_4_:Nd^3+^-PEG-Alk, and NaYF_4_:Yb^3+^/Er^3+^@NaYF_4_:Nd^3+^-PEG-RGDS nanoparticles on both Hep-G2 and HeLa cells was determined, confirming no adverse effect on their survival and proliferation. The interaction of the nanoparticles with Hep-G2 cells was monitored by confocal microscopy at both 808 and 980 nm excitation. The NaYF_4_:Yb^3+^/Er^3+^@NaYF_4_:Nd^3+^-PEG-RGDS nanoparticles were localized on the cell membranes due to specific binding of the RGDS peptide to integrins, in contrast to the NaYF_4_:Yb^3+^/Er^3+^@NaYF_4_:Nd^3+^-PEG-Alk particles, which were not engulfed by the cells. The NaYF_4_:Yb^3+^/Er^3+^@NaYF_4_:Nd^3+^-PEG-RGDS nanoparticles thus appear to be promising as a new non-invasive probe for specific bioimaging of cells and tissues. This development makes the nanoparticles useful for diagnostic and/or, after immobilization of a bioactive compound, even theranostic applications in the treatment of various fatal diseases.

## Introduction

Lanthanide-doped upconversion nanoparticles (UCNPs) have recently attracted a great deal of attention as promising materials for various biomedical applications including medical diagnostics, mainly for *in vitro* and *in vivo* imaging, but also in longer perspective for drug and gene delivery, and photothermal and photodynamic therapy of malignancies (Duan et al., 2018; Qin et al., 2019). The particles also find utilization in sensing applications, such as environmental hazard detection, food assays, and biological analysis (Chen et al., 2014; Poláchová et al., 2019; Zhang et al., 2019). Interest in the UCNPs comes from their superior optical properties, such as a narrow line emission, long luminescence lifetime, high photostability, and absence of background fluorescence interference (Wolfbeis, 2015). The main advantage of UCNPs is in their ability to convert low energy excitation photons (i.e., from NIR spectral range), which can penetrate deeper into biological tissues (up to 5 cm) (Li et al., 2017) than visible light, to high-energy photons, e.g., ultraviolet or visible, via anti-Stokes emission (Zhu et al., 2017). Moreover, UCNPs are characterized with low absorption and scattering rate. In contrast to the high energetic photons, NIR light is not harmful for the tissue and do not induce autofluoroscence, which provides significant contrast improvement.

Conventional UCNPs are mostly composed of an inorganic crystalline NaYF_4_ matrix doped by two types of lanthanide ions, one acting as an activator and another acting as a sensitizer (Auzel, 2004). Due to a large absorption cross-section, Yb^3+^ is typically used as a sensitizer responsible for the accumulation of excitation light energy and its transfer to the activator ion via non-radiative energy transfer (Bagheri et al., 2019). Yb^3+^ matches several activator ions, such as Tm^3+^, Er^3+^, or Ho^3+^, and in combination with them generates upconversion luminescence after absorption of NIR light at 980 nm (Zhou et al., 2015). Although excitation at 980 nm is very efficient, it is not beneficial for biomedical applications, especially for *in vivo* studies, because it overlaps with the absorption band of water, which can cause unwanted overheating and damage to cells and tissues (Zhan et al., 2011). Therefore, attention is recently focused on shifting the excitation of UCNPs to lower excitation wavelengths, where the absorption of water is minimal. It was found that when Nd^3+^ is codoped with Yb^3+^ and activator into a UCNP matrix, the nanoparticles can be excited at both 808 and 980 nm (Lai et al., 2014; Chen et al., 2015). Excitation of Nd^3+^-sensitized UCNPs at 808 nm benefits from minimal photothermal effects and provides deeper living tissue penetration compared to that of conventional 980 nm excitation (Wang et al., 2014; Wiesholler et al., 2019).

A broad range of synthetic strategies have been utilized to produce UCNPs with well-controlled nanoparticle size, composition, crystalline fidelity, and luminescence efficiency. Among these approaches, thermal decomposition, conventional and high-temperature coprecipitation, hydro(solvo)thermal or sol-gel synthesis, and preparation in the presence of ionic liquids have been extensively used (Liu et al., 2009; Wang et al., 2011; Naccache et al., 2015). Despite the substantial effort devoted to the synthesis, modification and characterization of UCNPs, their translation to the clinic is still far from complete, and several important issues need to be resolved before their introduction in praxis. For example, knowledge about the chemical stability and interactions between the particles and their polymer coatings is insufficient, as well as the behavior and colloidal stability of the surface-coated UCNPs in organism (Wilhelm, 2017). UCNPs disintegrate in highly diluted water dispersions, decreasing the upconversion luminescence intensity (Plohl et al., 2017). This process can be accompanied by the release of fluoride and lanthanide ions from the particles into the plasma or tissue, inducing cell death (Lahtinen et al., 2017). An additional problem consists of a relatively low upconversion luminescence efficiency and water-related quenching of the luminescence (Arppe et al., 2015). To address these difficulties, a dense and thick coating, preferably from an amphiphilic polymer, should be formed around the particles (Plohl et al., 2017). Modification of the UCNP surface with polymers affects not only the dispersibility in physiological media but also the chemical stability (preventing lanthanide ion leakage) and biocompatibility. Coating can be achieved by a ligand exchange, ligand oxidation, layer-by-layer technique, miniemulsion polymerization, self-assembly, or solvent evaporation technique (Muhr et al., 2014; Sedlmeier and Gorris, 2015). Nevertheless, the most frequently used inorganic coatings of UCNPs are still based on silica derivatives that can contain various reactive groups for the conjugation of drugs and other molecules. Strategies for silica coating typically include reverse microemulsion or the Stöber method (Stöber and Fink, 1968; Guerrero-Martinez et al., 2010). Different polymeric materials have also been suggested to change the hydrophobic UCNPs into hydrophilic UCNPs and to encapsulate biomolecules, including drugs. Such polymers include poly(acrylic acid), poly(ethylene glycol) (PEG) and its derivatives (e.g., PEG-neridronate), poly(vinylpyrrolidone), polyethyleneimine, poly(maleic anhydride-*alt*-1-octadecene), chitosan, dextran, etc. (Wilhelm et al., 2015; Gee and Xu, 2018; Mandl et al., 2019; Patsula et al., 2019).

The aim of this research was to develop biocompatible UCNP nanoparticles that absorb NIR light at both 808 and 980 nm excitation. Such particles might serve as a base for future development of UCNPs incorporating photoactivatable drugs, specifically those containing photosensitizers for photodynamic therapy of tumors. To achieve this goal, monodisperse spherical NaYF_4_:Yb^3+^/Er^3+^ cores were synthesized, and homogeneous NaYF_4_:Nd^3+^ shells of different thicknesses were deposited on them to (*i*) enhance the upconversion luminescence at 980 nm excitation and (*ii*) simultaneously shift the excitation to 808 nm due to the presence of Nd^3+^ ions in the shell. The particles were modified by bisphosphonate- and alkyne-terminated PEG with a strong binding affinity to lanthanides on the particle surface and an ability to conjugate biomolecules, e.g., azido-modified RGDS peptide via Cu(I) catalyzed click reaction, respectively. The cytotoxicity of unmodified, PEGylated, and RGDS-conjugated core-shell NaYF_4_:Yb^3+^/Er^3+^@NaYF_4_:Nd^3+^ nanoparticles was investigated using human cancer cell lines, hepatocellular carcinoma Hep-G2 cells, and cervical epithelioid carcinoma HeLa cells. The particle biodistribution in the cells was monitored by an inverted confocal fluorescence microscope with a laser at 980 nm excitation and a 140 fs pulse.

## Experimental

### Chemicals and Materials

Anhydrous neodymium(III), yttrium(III), ytterbium(III) and erbium(III) chlorides (99%), octadec-1-ene (90%), ammonium hydrogen difluoride, copper(II) sulfate, sodium L-ascorbate, Igepal CO-520 [polyoxyethylene(5) nonylphenyl ether], 2-amino-2-(hydroxymethyl)propane-1,3-diol (Tris), 2-[4-(2-hydroxyethyl)piperazin-1-yl]ethanesulfonic acid (HEPES), and phosphate-buffered saline (PBS) were obtained from Sigma-Aldrich (St. Louis, MO, USA). CellMask™ deep red was from Thermo Fisher Scientific (Waltham, MA, USA); α-NHS-ω-alkyne poly(ethylene glycol) (NHS-PEG_3,815_-Alk; *M*_w_ = 3,815 Da and NHS-PEG_5,475_-Alk; *M*_w_ = 5,475 Da) were purchased from Rapp Polymere (Tübingen, Germany). Oleic acid (OA), hexane, methanol, ethanol, and acetone were purchased from Lach-Ner (Neratovice, Czech Republic). Sodium neridronate and neridronate-PEG-alkyne (Ner-PEG-Alk) were prepared as described earlier (Kostiv et al., 2017c; Mickert et al., 2019). Azidopentanoyl-GGGRGDSGGGY-NH_2_ (RGDS) peptide was synthesized by the standard Fmoc/tBu solid-phase technique on a TentaGel® Rink-Amide-R resin according to previously published procedures (Proks et al., 2012; Kostiv et al., 2016). Dulbecco's modified Eagle's medium (DMEM) and fetal calf serum were purchased from Life Technologies (Carlsbad, CA, USA). Other reagent grade chemicals were obtained from commercial sources and used as received. Cellulose dialysis membranes (MWCO 0.5, 3.5, 14, and 100 kDa) were purchased from Spectrum Europe (Breda, Netherlands). Ultrapure Q-water ultrafiltered on a Milli-Q Gradient A10 system (Millipore; Molsheim, France) was used in all experiments.

### Synthesis of Core NaYF_4_:Yb^3+^/Er^3+^ Nanoparticles

Core NaYF_4_:Yb^3+^/Er^3+^ nanoparticles were synthesized according to previous publications with minor modifications (Kostiv et al., 2017a,b). In a 100-ml three-neck flask, a mixture of YCl_3_ (0.78 mmol), YbCl_3_ (0.2 mmol), ErCl_3_ (0.02 mmol), OA (6 ml), and octadec-1-ene (15 ml) was heated at 160°C for 30 min with stirring under Ar flow. After cooling to room temperature (RT), a solution of NaOH (4 mmol) and NH_4_F·HF (2.6 mmol) in methanol (5 ml) was added, and the mixture was slowly heated at 120°C under an Ar atmosphere until methanol evaporation; the reaction then continued at 300°C for 1.5 h. After cooling to RT, the resulting core NaYF_4_:Yb^3+^/Er^3+^ nanoparticles were collected by centrifugation (6,000 rpm; 30 min), redispersed in hexane (16 ml), precipitated by ethanol (10 ml), and separated by centrifugation (6,000 rpm; 30 min). The sedimentation-redispersion cycle was repeated two times and the particles were finally dispersed in hexane (10 mg/ml).

### Synthesis of Core-Shell NaYF_4_:Yb^3+^/Er^3+^@NaYF_4_:Nd^3+^ Nanoparticles

Core-shell NaYF_4_:Yb^3+^/Er^3+^@NaYF_4_:Nd^3+^ nanoparticles with 0.1, 0.3, 0.5, or 0.7 mmol of NaYF_4_:Nd^3+^ were synthesized according to an earlier procedure (Podhorodecki et al., 2018). Briefly, a mixture of YCl_3_ (0.08, 0.24, 0.4, or 0.56 mmol), NdCl_3_ (0.02, 0.06, 0.1, or 0.14 mmol), OA (6 ml), and octadec-1-ene (15 ml) was heated at 160°C for 30 min with stirring under an Ar atmosphere and cooled to RT. The core NaYF_4_:Yb^3+^/Er^3+^ nanoparticles (125 mg) in hexane (12.5 ml), NaOH (0.4, 1.2, 2, or 2.8 mmol), and NH_4_F·HF (0.26, 0.78, 1.3, or 1.82 mmol) in methanol were added, and the mixture was slowly heated to 70°C under an Ar atmosphere and then at 300°C for 1.5 h. The mixture was cooled to RT, and the resulting core-shell NaYF_4_:Yb^3+^/Er^3+^@NaYF_4_:Nd^3+^ nanoparticles were washed with hexane and ethanol as described above and dispersed in hexane (10 mg/ml). In further experiments, NaYF_4_:Yb^3+^/Er^3+^@NaYF_4_:Nd^3+^ nanoparticles with 0.5 mmol of NaYF_4_:Nd^3+^ were used due to their uniformity and high luminescence efficiency at both 808 and 980 nm excitation.

### Synthesis of NaYF_4_:Yb^3+^/Er^3+^-PEG-Alk, NaYF_4_:Yb^3+^/Er^3+^@NaYF_4_:Nd^3+^-PEG-Alk, and NaYF_4_:Yb^3+^/Er^3+^@NaYF_4_ :Nd^3+^-PEG-RGDS Nanoparticles

The surface of the core NaYF_4_:Yb^3+^/Er^3+^ and core-shell NaYF_4_:Yb^3+^/Er^3+^@NaYF_4_:Nd^3+^ nanoparticles (containing 0.5 mmol of NaYF_4_:Nd^3+^) were modified by Ner-PEG-Alk of two different molecular weights, i.e., Ner-PEG_3,815_-Alk and Ner-PEG_5,475_-Alk. Prior to the modification, excess OA ligands were removed by washing the particles with hexane/ethanol (1/1 v/v), ethanol, ethanol/water (1/1 v/v), and water during five sedimentation-redispersion cycles; finally, the particles were dialyzed (MWCO = 14 kDa) against water. Ner-PEG_3,815_-Alk (6 mg) or Ner-PEG_5,475_-Alk (8 mg) was added to an aqueous particle dispersion (5 ml; 4 mg/ml), and the mixture was stirred at RT for 12 h. Excess PEG was removed by dialysis against water using a cellulose membrane (MWCO = 14 kDa) at RT for 48 h; the water (2.5 l) was exchanged two times. For biological experiments, the NaYF_4_:Yb^3+^/Er^3+^@NaYF_4_:Nd^3+^-PEG_5,475_-Alk nanoparticles were selected due to a high content of Ner-PEG_5,475_-Alk bound to the particle surface, as confirmed by TGA analysis.

NaYF_4_:Yb^3+^/Er^3+^@NaYF_4_:Nd^3+^-PEG_5,475_-Alk nanoparticles were conjugated with azidopentanoyl-GGGRGDSGGGY-NH_2_ peptide via a click reaction. Briefly, an aqueous NaYF_4_:Yb^3+^/Er^3+^@NaYF_4_:Nd^3+^-PEG_5,475_-Alk particle dispersion (1 ml; 20 mg/ml), azidopentanoyl-GGGRGDSGGGY-NH_2_ peptide (20 μl; 1 mg/ml), and sodium ascorbate (20 μl; 20 μg/ml) were purged with Ar for 30 min, and aqueous 0.05 M CuSO_4_ solution (10 μl) was added, and the mixture was purged with Ar for an additional 30 min. The resulting NaYF_4_:Yb^3+^/Er^3+^@NaYF_4_:Nd^3+^-PEG-RGDS nanoparticle dispersion was sonicated for 10 min and washed by dialysis against water (MWCO = 100 kDa) at RT for 48 h; the water (2.5 l) was exchanged two times.

### Characterization of Nanoparticles

The morphology, elemental composition, and crystal structure of the nanoparticles were analyzed using a Tecnai Spirit G2 transmission electron microscope (TEM; FEI; Brno, Czech Republic). Nanoparticles were deposited on a standard carbon-coated copper grid and characterized by three TEM modes at 120 kV. (*i*) The morphology of the nanoparticles was visualized by bright field imaging (BF), (*ii*) the elemental composition was obtained from energy-dispersive X-ray (EDX) analysis (EDAX detector; Mahwah, NJ, USA), and (*iii*) the crystal structure was verified by means of selected area electron diffraction (SAED). The nanoparticle size and distribution were determined by measuring at least 300 nanoparticles from TEM micrographs using ImageJ software. The number-average diameter (*D*_n_), weight-average diameter (*D*_w_) and uniformity (dispersity Ð) were calculated as follows:

(1) $$\begin{array}{l} D_{\text{n}}=\sum {\text{N}}_{\text{i}}D_{\text{i}}/\sum {\text{N}}_{\text{i}}, \end{array}$$

(2) $$\begin{array}{l} D_{\text{w}}=\sum {\text{N}}_{\text{i}}D_{\text{i}}^{4}/\sum {\text{N}}_{\text{i}}D_{\text{i}}^{3}, \end{array}$$

(3) $$\begin{array}{l} Ð=D_{\text{w}}/D_{\text{n}}, \end{array}$$

where N_i_ and *D*_i_ are the number and diameter of the nanoparticles, respectively. The elemental composition was calculated from TEM/EDX spectra by EDAX software and post-processed in the common MS Excel spreadsheet program. Elements (C and Cu) originating from the supporting carbon-coated copper TEM grid and possible impurities (small Si and O peaks originating from dust) were subtracted, and the remaining elements were normalized to 100%. The TEM/SAED patterns were processed with ProcessDiffraction software (Lábár, 2005) and compared to the theoretical X-ray diffraction patterns calculated with PowderCell software (Kraus and Nolze, 1996); the crystal structures for calculation of diffraction patterns were obtained from the freeware Crystallography Open Database (Glasser, 2016).

The hydrodynamic particle diameter (*D*_h_), size distribution (polydispersity *PD*), and ζ-potential were determined by dynamic light scattering (DLS) on a ZEN 3600 Zetasizer Nano Instrument (Malvern Instruments; Malvern, UK). The particle dispersion was measured at 25°C, and *D*_h_ and *PD* were calculated from the intensity-weighted distribution function obtained by CONTIN analysis of the correlation function embedded in Malvern software.

ATR FTIR spectra were recorded on a Nexus Nicolet 870 FTIR spectrometer (Madison, WI, USA) equipped with a liquid nitrogen-cooled mercury cadmium telluride detector and a Golden Gate single reflection ATR cell (Specac; Orpington, UK). Thermogravimetric analysis (TGA) of the particles was performed in air in the temperature range 30–600°C at a heating rate of 10°C/min using a PerkinElmer Pyris 1 thermogravimetric analyzer (Shelton, CT, USA).

Upconversion luminescence spectra of nanoparticles in a hexane (2 ml; 1 mg/ml), hexane/water or hexane/D_2_O (2 ml/10 μl) emulsion stabilized with Igepal CO-520 (100 μl) were measured using an FS5 spectrofluorometer (Edinburgh Instruments; Edinburgh, UK) coupled with a CW 980 nm laser diode as an excitation source (MDL-III-980-2W; output laser power 2 W and beam size 5 × 8 mm^2^). The particle dispersion in hexane was stirred prior to the measurement, while the hexane/water or hexane/D_2_O emulsions were sonicated with a USC 300 THD/HF ultrasonic bath (VWR; Lutterworth, UK) at RT for 10 min.

A custom-build experimental setup was used for measurements of the optical spectra of UCNPs excited by two CW fiber-coupled infrared diode lasers with nominal power 2 W (MDL-III-808/MDL-III-980; CNI Optoelectronics; Changchun, China) and emission wavelengths of 808 and 980 nm. A multimode fiber with core diameter 1 mm was used to homogenize both laser beams and ensure the same excitation beam profile focused on the sample. Laser light was collimated and focused at the center of the sample cuvette with an achromatic lens of focal length of 35 mm (Edmund Optics; Barrington, NJ, USA) to excite a large volume of the particle dispersion. The resulting beam waist was measured using a BP209-VIS scanning-slit optical beam profiler (Thorlabs; Newton, NH, USA). Due to the use of achromatic doublets, the beam waist at the focus was almost identical for both lasers. The values of the fitted waists equaled 1,260 ± 20 and 1,280 ± 20 μm for the 808 nm and 980 nm lasers, respectively, which corresponds to an average power density of ~30 W/cm^2^. The head of a FieldMax II thermal power sensor (Coherent; Palo Alto, CA, USA) was positioned 5 mm behind the cuvette rear wall to monitor the laser power during the experiment. Luminescence spectra were collected with a dry microscope objective (NA 0.12) equipped with a short-pass F38-749 filter (Semrock; Rochester, NY, USA) and focused on the multimode fiber attached to a Flame VIS spectrometer (Ocean Optics; Largo, FL, USA). Background-corrected spectra were collected with an acquisition time of 2.5 s.

### Cell Cultivation and Monitoring

Human hepatocellular carcinoma Hep-G2 (ECACC 85011430) and human cervix epitheloid carcinoma HeLa cells (ECACC 93021013) were cultivated at 37°C in DMEM with 3 mM glutamine, 10% (v/v) fetal calf serum, 10 mM HEPES, 100 IU/ml penicillin, 100 μg/ml streptomycin, and 5 mM glucose in humidified air with 5 % CO_2_. The cells were then cultured on poly(L-lysine)-coated glass coverslips in DMEM (2 ml) for 2 d, incubated with a nanoparticle dispersion (20, 40, and 150 μl; 5 mg/ml) for 24 h, transferred to a thermostable chamber at 37°C under 5% CO_2_ atmosphere, mimicking normal cultivation conditions, and finally observed in a Leica TCS SP8 AOBS confocal inverted fluorescence microscope (Wetzlar, Germany) equipped with an objective HC PL APO 63 ×/1.20 NA W CORR CS2, WD = 0.3 mm. Particles were excited by a Chameleon Ultra I pulsed infrared tunable laser with wavelength range 690–1,040 nm, maximum output power 4 W, pulse frequency 80 MHz, pulse width ~140 fs and laser intensity controlled by electrooptical EOM modulator (Coherent; Santa Clara, CA, USA) and attenuator at 980 nm excitation.

CellMask™ deep red-stained cell plasma membrane without the cell type-specific differences exhibited by lectins was visualized in a standard fluorescence confocal microscope with excitation and emission at 649 and 666 nm, respectively. A WLL2 supercontinuous pulsed laser for two-photon excitation (NKT Photonics; Birkerød, Denmark) was used for excitation at 470–670 nm with an average laser power ~1.5 mW. The z-planes of the cells and nanoparticles were viewed by z-scan mode of the microscope with excitation at 808 and 980 nm. Overlaps of image planes showed the distribution of nanoparticles in the cell or their binding to the plasma membrane using λ-scan mode.

### Cell Toxicity

Hep-G2 and HeLa cells (both 5 × 10^4^) were cultured for 24 h as described above and incubated with NaYF_4_:Yb^3+^/Er^3+^@NaYF_4_:Nd^3+^, NaYF_4_:Yb^3+^/Er^3+^@NaYF_4_:Nd^3+^-PEG-Alk, and NaYF_4_: Yb^3+^/Er^3+^@PEG-RGDS nanoparticles (0.001–1 mg per ml of DMEM) at 37°C for 72 h under a 5% CO_2_ atmosphere. *In vitro* cell viability was determined by 0.4% trypan blue staining (Thermo Fisher Scientific), and the fraction of living cells was counted on a Countess automated cell counter (Thermo Fisher Scientific).

## Results and Discussion

### Synthesis and Structure of NaYF_4_:Yb^3+^/Er^3+^ Core and NaYF_4_:Yb^3+^/Er^3+^@NaYF_4_:Nd^3+^ Core-Shell Nanoparticles

Both the starting NaYF_4_:Yb^3+^/Er^3+^ core and NaYF_4_:Yb^3+^/Er^3+^@NaYF_4_:Nd^3+^ core-shell nanoparticles were synthesized in a high-boiling organic solvent (octadec-1-ene) in the presence of oleic acid (OA) as a stabilizer by high-temperature coprecipitation of lanthanide chlorides with different amounts of shell precursors.

**Figure 1:**
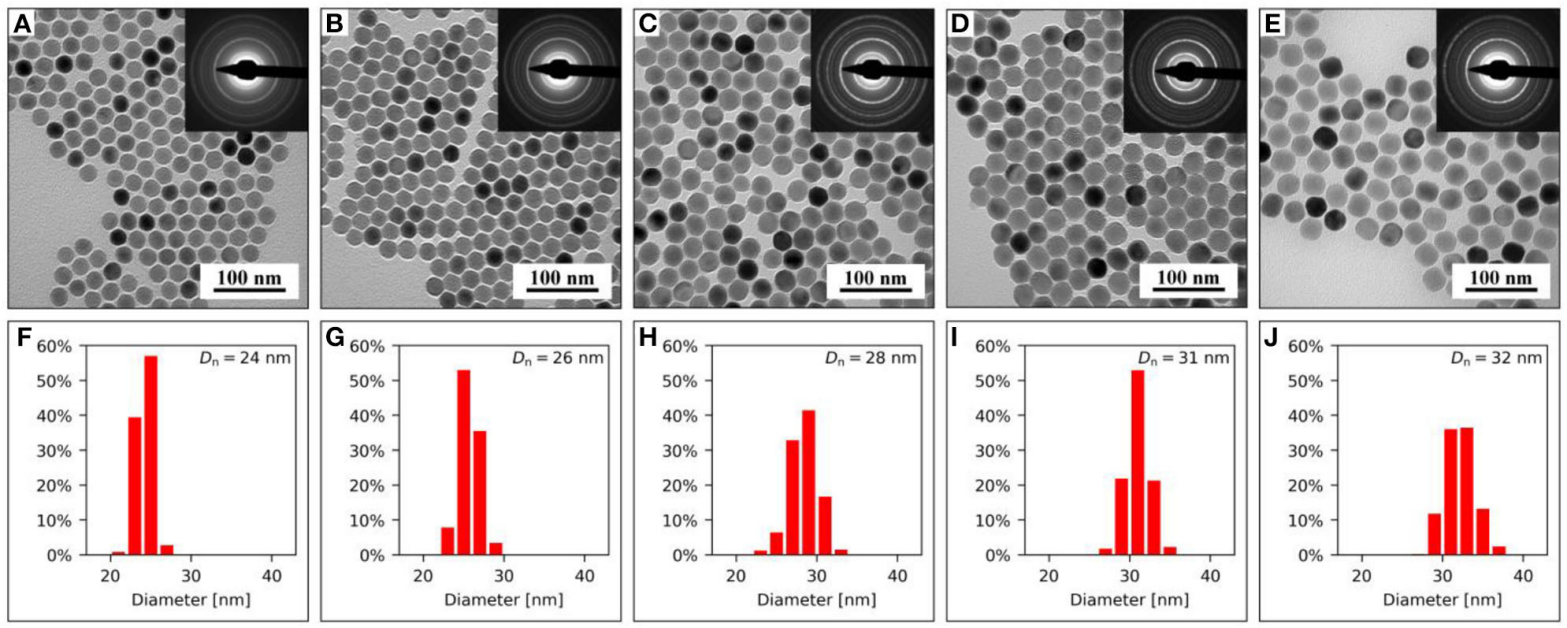
**(A–E)** TEM/BF micrographs with TEM/SAED diffraction patterns (insets) and **(F–J)** corresponding particle size distributions of NaYF_4_:Yb^3+^/Er^3+^@NaYF_4_:Nd^3+^ nanoparticles containing **(A,F)** 0, **(B,G)** 0.1, **(C,H)** 0.3, **(D,I)** 0.5, and **(E,J)** 0.7 mmol of NaYF_4_:Nd^3+^. Number-average particle diameters *D*_n_ are shown in the upper right corners of the histograms.

**Figure 2:**
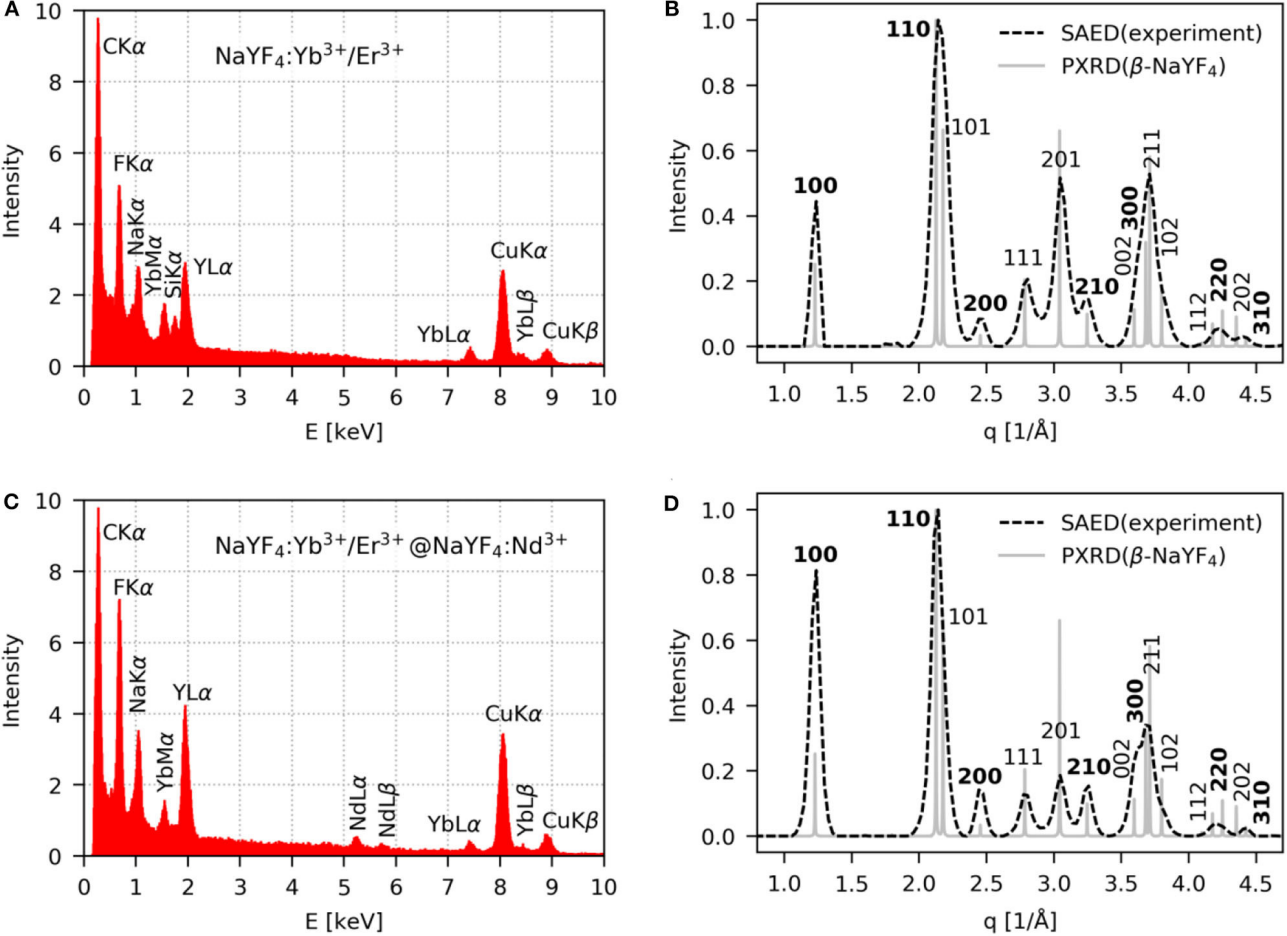
**(A,C)** TEM/EDX and **(B,D)** TEM/SAED analysis of **(A,B)** NaYF_4_:Yb^3+^/Er^3+^ and **(C,D)** NaYF_4_:Yb^3+^/Er^3+^@NaYF_4_:Nd^3+^(0.5 mmol) nanoparticles. Experimental TEM/SAED diffractograms (dashed lines) were compared with theoretically calculated PXRD diffractograms for the hexagonal phase of β-NaYF_4_ (full lines).

The NaYF_4_:Yb^3+^/Er^3+^ core was composed of an optically inert NaYF_4_ host matrix and optically active sensitizer (Yb^3+^) and activator (Er^3+^), enabling excitation at 980 nm. The size distribution and average size of the nanoparticles were determined from the TEM/BF micrographs (Figure 1). The cores were spherical in shape with number-average diameter *D*_n_ = 24.2 ± 0.9 nm and high uniformity (Ð = 1; Figures 1A,F). These cores served as seeds for the subsequent synthesis of differently thick NaYF_4_:Nd^3+^ shells to introduce a second sensitizer (Nd^3+^) into the crystal matrix. The shell played two important roles: (*i*) it prevented surface quenching by passivation of optically active ions on the cores due to enhanced upconversion emission intensity and (*ii*) the presence of the Nd^3+^ sensitizer in the core-shell matrix enabled excitation of the particles at both 808 and 980 nm. The resulting NaYF_4_:Yb^3+^/Er^3+^@NaYF_4_:Nd^3+^ core-shell nanoparticles were isometric and monodisperse (Ð < 1.02). The shell thickness was controlled by the amount of Y and Nd precursors, NaOH, and NH_4_F·HF used in the synthesis. With increasing concentrations of the abovementioned precursors in the reaction feed and constant amounts of NaYF_4_:Yb^3+^/Er^3+^ seeds, the NaYF_4_:Nd^3+^ shell became thicker, the average particle size became larger (*D*_n_ = 26–32 nm; Figures 1B–E,G–J; Table S1), and the Nd content in the particles increased, as determined by TEM/EDX analysis (Figure S1). The crystalline structure corresponding to the hexagonal β-NaYF_4_ phase was the same in all synthesized particles, as indicated by the very similar TEM/SAED patterns (Figure 1, insets in the TEM/BF micrographs). The diffraction positions were the same, and only their intensities slightly changed, which could be attributed to increasing preferred orientation, as will be discussed below. Representative TEM/EDX spectra and TEM/SAED analyses of starting NaYF_4_:Yb^3+^/Er^3+^ and NaYF_4_:Yb^3+^/Er^3+^@NaYF_4_:Nd^3+^ core-shell particles containing 0.5 mmol NaYF_4_:Nd^3+^ confirmed the expected elemental composition of the nanoparticles (Figure 2). The TEM/EDX spectrum of NaYF_4_:Yb^3+^/Er^3+^ nanoparticles showed strong peaks of the main Na, Y, and F elements, a weaker Yb peak, and C and Cu peaks from the standard supporting carbon-coated copper TEM grid (Figure 2A). Two smaller peaks (Si and O at 1.74 and 0.52 eV, respectively) were attributed to a small amount of impurities, probably dust; the concentration of Si was <0.5%, and the content of Er was below the detection limit of the measurement. The spectrum of the NaYF_4_:Yb^3+^/Er^3+^@NaYF_4_:Nd^3+^ nanoparticles (0.5 mmol of NaYF_4_:Nd^3+^) contained additional Nd peaks and almost no signs of impurities (negligible amount of Si; Figure 2C). Note that the heights of the C and Cu peaks in the TEM/EDX spectra were somewhat arbitrary, depending on the relative amount of nanoparticles and the vicinity of the Cu mesh at a given location on the TEM grid. The experimental TEM/SAED diffraction patterns (Figures 2B,D; dashed lines) were compared to the theoretically calculated powder X-ray diffraction (PXRD) patterns of β-NaYF_4_ (Figures 2B,D; full lines). Perfect agreement between the SAED and PXRD diffraction positions confirmed that all particles exhibited a hexagonal β-NaYF_4_ phase structure. Differences among the diffraction intensities of the two particle types and the theoretically calculated PXRD diffractogram of β-NaYF_4_ could be attributed to the preferred orientation. The smallest NaYF_4_:Yb^3+^/Er^3+^ nanocrystals were spherical, with random orientation, and, consequently, they exhibited almost the same SAED and PXRD diffraction intensities (Figure 2B). The larger NaYF_4_:Yb^3+^/Er^3+^@NaYF_4_:Nd^3+^ nanocrystals with 0.5 mmol of NaYF_4_:Nd^3+^ were slightly faceted, with a tendency to lie on the facets; as a result, their orientation on the supporting carbon film was no longer random. The two strongest diffractions were (100) and (110), suggesting a preferred orientation with zone axis [*uvw*] = [001] (Figure 2D); calculation of the zone axis from a pair of strong diffractions has been described elsewhere (Andrews et al., 1967; Beeston et al., 1972). The strongest peaks on the diffraction pattern should obey the Weiss zone law (WZL): *hu* + *kv* + *lw* = 0, where (*h, k, l*) are the diffraction indices of the planes, and [*u, v, w*] are the indices of the zone axis (Andrews et al., 1967). If the zone axis [*uvw*] = [001], the WZL takes a simple form *l* = 0, which means that the strongest diffractions should be of the (*hk*0) type. The diffractions of this type are marked in bold font in Figures 2B,D. For the core NaYF_4_:Yb^3+^/Er^3+^ nanocrystals, the intensity of (*hk*0) diffraction was comparable to that of other diffractions; the small intensity discrepancies could be attributed to the intrinsic differences between the SAED and PXRD methods and possible experimental errors (Figure 2B). For the NaYF_4_:Yb^3+^/Er^3+^@NaYF_4_:Nd^3+^ core-shell nanoparticles with 0.5 mmol of NaYF_4_:Nd^3+^, the (*hk*0) diffractions were stronger than both the theoretically calculated PXRD intensities (note that the SAED and PXRD diffractograms were normalized to the intensity of the strongest (110) diffraction) and experimentally determined SAED intensities of other diffractions of the general type (*hkl*). This consistent result confirmed the correctness of the TEM/SAED analysis. The enhanced relative intensity of the (*hk*0) diffractions corresponded to an increasing trend of forming faceted nanocrystals at higher Nd concentrations (Figure 1).

### Surface Engineering of NaYF_4_:Yb^3+^/Er^3+^ and NaYF_4_:Yb^3+^/Er^3+^@NaYF_4_:Nd^3+^ Nanoparticles

The starting NaYF_4_:Yb^3+^/Er^3+^ core and NaYF_4_:Yb^3+^/Er^3+^@NaYF_4_:Nd^3+^ core-shell nanoparticles stabilized by OA are well-dispersible in a non-polar medium, e.g., hexane. However, before nanoparticle modification, to make them dispersible in aqueous media, as required for biological and/or medical applications, OA has to be removed from the surface by thorough washing with ethanol and water. After washing, the hydrodynamic diameters of the NaYF_4_:Yb^3+^/Er^3+^ core and NaYF_4_:Yb^3+^/Er^3+^@NaYF_4_:Nd^3+^ core-shell particles in water were 185 and 195 nm, respectively, with rather low polydispersity (*PD* = 0.16 and 0.18; Figures S2a,b). This observation was in agreement with the monodispersity of NaYF_4_:Yb^3+^/Er^3+^ and NaYF_4_:Yb^3+^/Er^3+^@NaYF_4_:Nd^3+^ particles in water measured by TEM/BF (Figures S3a,c,e,g). Note that the nanoparticle size and shape were not affected by washing. It is not surprising that *D*_h_ > *D*_n_, as the former was measured in water and represented an intensity-based mean diameter that is sensitive to the presence of large particles, while the latter was sensitive to small particles and was analyzed in a dry state; moreover, a partial particle aggregation in water could also increase the size. The surface charge of both core and core-shell particles was positive (ζ = 37 and 41 mV, respectively) due to the presence of lanthanide atoms on the surface. Such a high ζ-potential supports particle repulsion, ensuring good colloidal stability in water; let us note that particles with ζ-potential <30 mV typically have a tendency to aggregate (Lowry et al., 2016). Though the purified NaYF_4_:Yb^3+^/Er^3+^ and NaYF_4_:Yb^3+^/Er^3+^@NaYF_4_:Nd^3+^ particles were well-dispersible in water, they irreversibly aggregated in PBS used in biomedical applications. Therefore, we modified the NaYF_4_:Yb^3+^/Er^3+^ and NaYF_4_:Yb^3+^/Er^3+^@NaYF_4_:Nd^3+^ nanoparticle surfaces with both Ner-PEG_3,815_-Alk and Ner-PEG_5,475_-Alk. The size and shape of the modified nanoparticles remained the same as those measured by TEM/BF (Figures S3b,d,f,h). After modification with Ner-PEG_3,815_-Alk, the hydrodynamic particle diameter of NaYF_4_:Yb^3+^/Er^3+^ and NaYF_4_:Yb^3+^/Er^3+^@NaYF_4_:Nd^3+^ in water decreased from 185 and 195 nm to 86 and 110 nm, respectively, with *PD* = 0.14 and 0.13 (Figures S2c,e); if the particles were modified with Ner-PEG_5,475_-Alk, their *D*_h_ reached 92 and 116 nm, respectively, with *PD* = 0.12 (Figures S2d,f). The ζ-potential of the NaYF_4_:Yb^3+^/Er^3+^-PEG_3,815_-Alk and NaYF_4_:Yb^3+^/Er^3+^-PEG_5,475_-Alk particles decreased to 9 and 5 mV, respectively, while that of NaYF_4_:Yb^3+^/Er^3+^@NaYF_4_:Nd^3+^-PEG_3,815_-Alk and NaYF_4_:Yb^3+^/Er^3+^@NaYF_4_:Nd^3+^-PEG_5,475_-Alk particles was only 2 and 4 mV, respectively. The nearly neutral surface charge of the PEGylated UCNPs, compared to the highly positive unmodified nanoparticles, indicated successful surface modification of the particles by PEG.

The colloidal stability of the particles was further investigated in 0.01 M PBS buffer used in experiments with cells and animals. Here, aggregation of the starting NaYF_4_:Yb^3+^/Er^3+^ core and NaYF_4_:Yb^3+^/Er^3+^@NaYF_4_:Nd^3+^ core-shell nanoparticles was due to the chelation of phosphates to lanthanides exposed on the particle surface and the relatively high ionic strength of PBS compared to water. In contrast, the colloidal stability of PEGylated nanoparticles in PBS remained similar to that in water, reaching *D*_h_ = 90 and 126 nm with *PD* = 0.13 and 0.15 for NaYF_4_:Yb^3+^/Er^3+^-PEG_3,815_-Alk and NaYF_4_:Yb^3+^/Er^3+^@NaYF_4_:Nd^3+^-PEG_3,815_-Alk, respectively (Figures S2c,e). According to the DLS measurements, the NaYF_4_:Yb^3+^/Er^3+^-PEG_5,475_-Alk and NaYF_4_:Yb^3+^/Er^3+^@NaYF_4_:Nd^3+^-PEG_5,475_-Alk particles had *D*_h_ = 93 and 129 nm, respectively, with *PD* = 0.13 (Figures S2d,f). This result shows that modification by Ner-PEG-Alk endowed the UCNPs with colloidal stability even in PBS due to the strong binding of neridronate to their surface and highly efficient steric stabilization by PEG.

The presence of functional alkyne groups on the UCNP surface is necessary to facilitate efficient conjugation of targeting moieties, such as peptides, vitamins, or antibodies. In this report, arginine-glycine-aspartic acid (RGD) peptide was used as a model biomolecule that was conjugated to the nanoparticle surface via copper (I)-catalyzed azide-alkyne 1,3-cycloaddition (click reaction). Compared to conventional bioconjugation reactions, the click reaction is beneficial due to its high selectivity, high reaction yield, and use of ambient reaction conditions, i.e., RT and aqueous media. After conjugation, the resulting NaYF_4_:Yb^3+^/Er^3+^@NaYF_4_:Nd^3+^-PEG_5,475_-RGDS nanoparticles were colloidally stable in water and 0.01 M PBS, reaching *D*_h_ = 116 and 130 nm, respectively, with PD = 0.14 and 0.13 (Figure S2g).

To further confirm the particle surface modification, ATR FTIR spectra of the unmodified and PEGylated NaYF_4_:Yb^3+^/Er^3+^ core and NaYF_4_:Yb^3+^/Er^3+^@NaYF_4_:Nd^3+^ core-shell particles were acquired (Figures S4a,b). The spectrum of the unmodified nanoparticles displayed characteristic bands of oleyl groups at 2,927 and 2,854 cm^−1^ attributed to asymmetric and symmetric CH_2_ stretching vibrations (Shukla et al., 2003). The intensity of the characteristic OA bands was low, especially in the spectrum of the NaYF_4_:Yb^3+^/Er^3+^@NaYF_4_:Nd^3+^ core-shell nanoparticles (Figure S4b, black line), due to the removal of OA adsorbed during the synthesis, thus confirming the efficacy of the washing procedure. After particle surface modification by Ner-PEG_3,815_-Alk and Ner-PEG_5,475_-Alk, the FTIR spectra revealed strong peaks at 2,886, 1,467, 1,342, 1,284, 1,112, 960, and 844 cm^−1^ (Figures S4a,b), which confirmed the presence of PEG on both the NaYF_4_:Yb^3+^/Er^3+^ core and NaYF_4_:Yb^3+^/Er^3+^@NaYF_4_:Nd^3+^ core-shell nanoparticles, in agreement with analogous modification of silver nanoparticles by PEG (Shameli et al., 2012). Although all the particles were lyophilized after washing and dialysis, residual water was still adsorbed on the particles, as the ATR FTIR spectra contained broad peaks of water O-H stretching vibrations in the 3,700–3,100 cm^−1^ region. The intensities of the water bands were low compared to the intensities of the other bands.

TGA of both unmodified and PEG_3,815_- or PEG_5,475_-coated NaYF_4_:Yb^3+^/Er^3+^ core and NaYF_4_:Yb^3+^/Er^3+^@NaYF_4_:Nd^3+^ core-shell nanoparticles was used to determine weight changes due to polymer decomposition at temperatures ranging from 25 to 600°C (Figures S4c,d). Residual water that was evaporated up to 150°C amounted to 0.5–5 wt.%, while OA on the NaYF_4_:Yb^3+^/Er^3+^ core and NaYF_4_:Yb^3+^/Er^3+^@NaYF_4_:Nd^3+^ core-shell nanoparticles decomposed at 300–400°C, reaching 3.5 and 2 wt.%, respectively. Thermal decomposition of PEG on the PEGylated particles started at 200°C and was completed at 500°C. The weight loss corresponded to 15 and 20 wt.% PEG_3,815_ or 21 and 25 wt.% PEG_5,475_ in the NaYF_4_:Yb^3+^/Er^3+^-PEG_3,815_-Alk core and NaYF_4_:Yb^3+^/Er^3+^@NaYF_4_:Nd^3+^-PEG_3,815_-Alk core-shell or NaYF_4_:Yb^3+^/Er^3+^-PEG_5,475_-Alk core and NaYF_4_:Yb^3+^/Er^3+^@NaYF_4_:Nd^3+^-PEG_5,475_-Alk core-shell nanoparticles, respectively. This result means that more PEG molecules were bound on the NaYF_4_:Yb^3+^/Er^3+^@NaYF_4_:Nd^3+^-PEG-Alk core-shell nanoparticles than on the NaYF_4_:Yb^3+^/Er^3+^-PEG-Alk core particles (by ~5 wt.%), which can be explained by size effects. Moreover, PEG_5,475_ was superior to PEG_3,815_ in terms of efficient particle surface modification, and therefore the NaYF_4_:Yb^3+^/Er^3+^@NaYF_4_:Nd^3+^-PEG_5,475_-Alk core-shell nanoparticles were preferred in the subsequent biological experiments.

### Cytotoxicity of Particles

**Figure 3:**
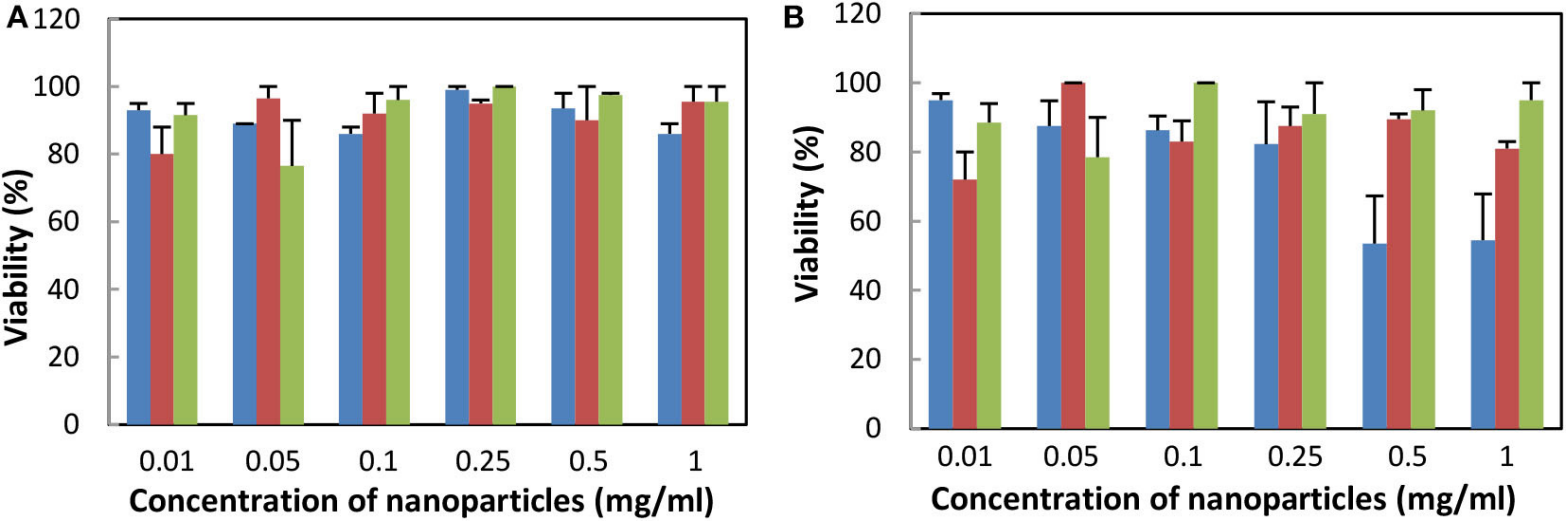
Dependence of viability of **(A)** Hep-G2 and **(B)** HeLa cells on the concentration of NaYF_4_:Yb^3+^/Er^3+^@NaYF_4_:Nd^3+^ (blue), NaYF_4_:Yb^3+^/Er^3+^@NaYF_4_:Nd^3+^-PEG_5,475_-Alk (red), and NaYF_4_:Yb^3+^/Er^3+^@NaYF_4_:Nd^3+^-PEG_5,475_-RGDS nanoparticles (green) using the trypan blue exclusion test. All differences between data were statistically non-significant, except for blue vs. other two bars at concentration 0.5 and 1 mg/ml for Hela cells, where *P* < 0.01.

As the developed UCNPs are prospectively intended for photodynamic therapy of tumors, Hep-G2 and HeLa cancer cell lines were selected for cytotoxicity investigation using a trypan blue exclusion test (Figure 3). Trypan blue, a hydrophilic tetrasulfonated anionic dye containing two azo chromophores, is commonly used to selectively detect dead cells. Incubation and/or penetration of NaYF_4_:Yb^3+^/Er^3+^@NaYF_4_:Nd^3+^, NaYF_4_:Yb^3+^/Er^3+^@NaYF_4_:Nd^3+^-PEG_5,475_-Alk, and NaYF_4_: Yb^3+^/Er^3+^@NaYF_4_:Nd^3+^-PEG_5,475_-RGDS nanoparticles (0–1 mg/ml) in the cells for 72 h virtually did not decrease their viability. The possible viability variations were within statistical error. Decrease of HeLa cell viability was observed only at a high concentration of the NaYF_4_:Yb^3+^/Er^3+^@NaYF_4_:Nd^3+^ particles (>0.5 mg/ml). Amounts of the nanoparticles >1 mg/ml could affect dilution of the culture medium, possibly skewing the results of the cytotoxicity assay. The results were comparable with those from previous studies (Kostiv et al., 2015), demonstrating the biocompatibility of the particles in the above concentration range. For further cell monitoring, particle concentrations that did not affect Hep-G2 viability were used.

### Upconversion Photoluminescence of NaYF_4_:Yb^3+^/Er^3+^ Core and NaYF_4_:Yb^3+^/Er^3+^@NaYF_4_:Nd^3+^ Core-Shell Nanoparticles at 808 and 980 nm Excitation

To investigate the optical properties of the NaYF_4_:Yb^3+^/Er^3+^ core and NaYF_4_:Yb^3+^/Er^3+^@NaYF_4_:Nd^3+^ core-shell nanoparticles, their upconversion photoluminescence spectra were measured at both 808 and 980 nm excitation wavelengths. Due to colloidal instability and sedimentation of the particles in hexane, a hexane/water emulsion stabilized with Igepal CO-520 was prepared for optical analysis. The particles dispersed in hexane quickly settled down, which decreased the intensity of the detected light. For example, the luminescence intensity of NaYF_4_:Yb^3+^/Er^3+^@NaYF_4_:Nd^3+^ (0.5 mmol) core-shell nanoparticles in hexane (1 mg/ml) decreased by 10-fold after 30 min, while nanoparticles dispersed in hexane/water emulsion stabilized with Igepal CO-520 exhibited similar luminescence intensities during the experiments (Figure S5). Therefore, all the photoluminescence spectra were measured in hexane/water emulsion with a concentration of 1 mg of particles/ml and a power density of ~30 W/cm^2^ at both 808 and 980 nm excitation wavelengths. Lasers were warmed-up for 30 min prior measurements and the laser power was monitored continuously during the experiment. To ensure that the luminescence intensity was not affected by variations in the particle concentration, spectra of all samples were measured in triplicate.

**Figure 4:**
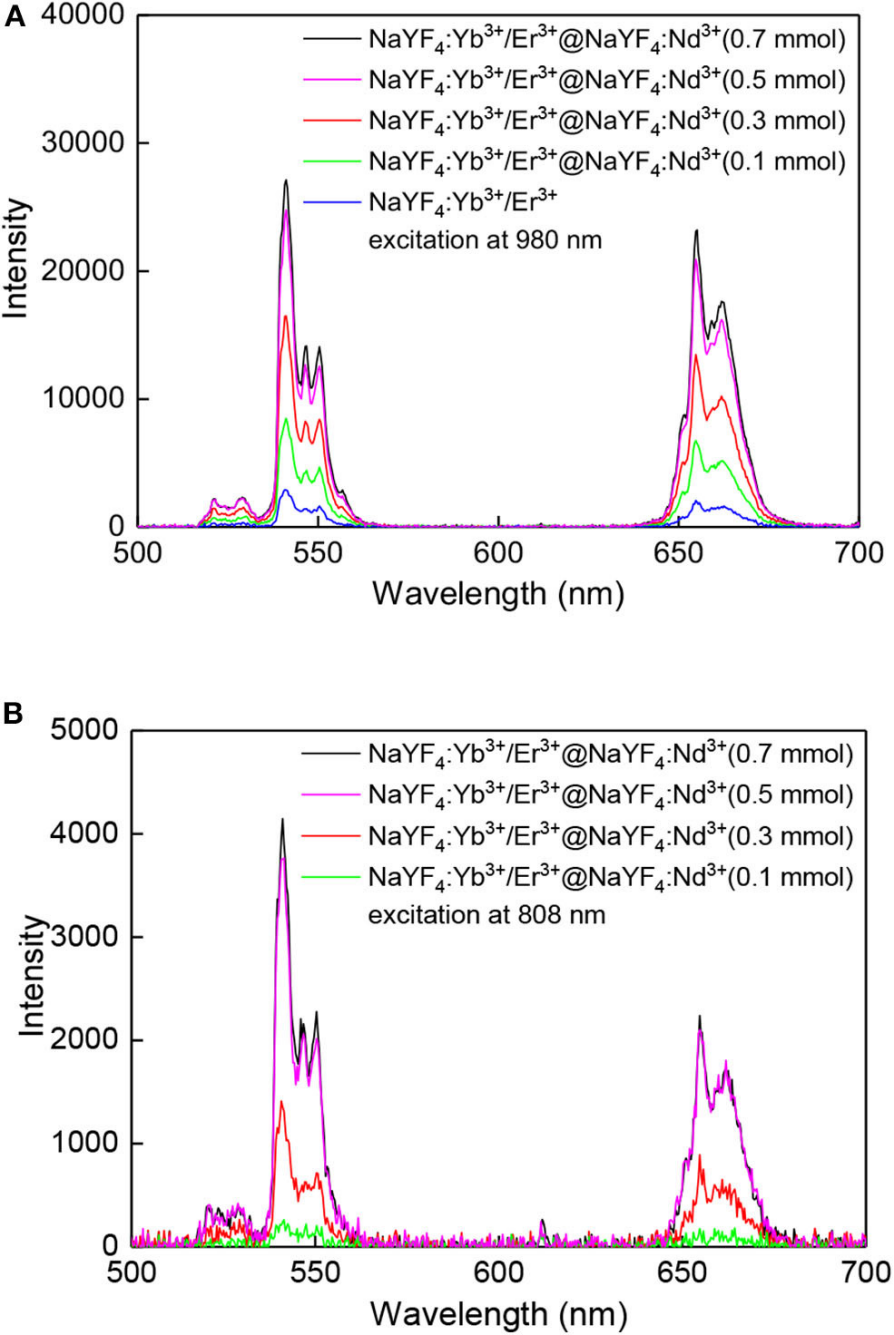
Upconversion photoluminescence spectra of nanoparticles (1 mg/ml) in hexane/water emulsion stabilized with Igepal CO-520 at **(A)** 980 and **(B)** 808 nm excitation; average power density of both lasers ~30 W/cm^2^.

**Figure 5:**
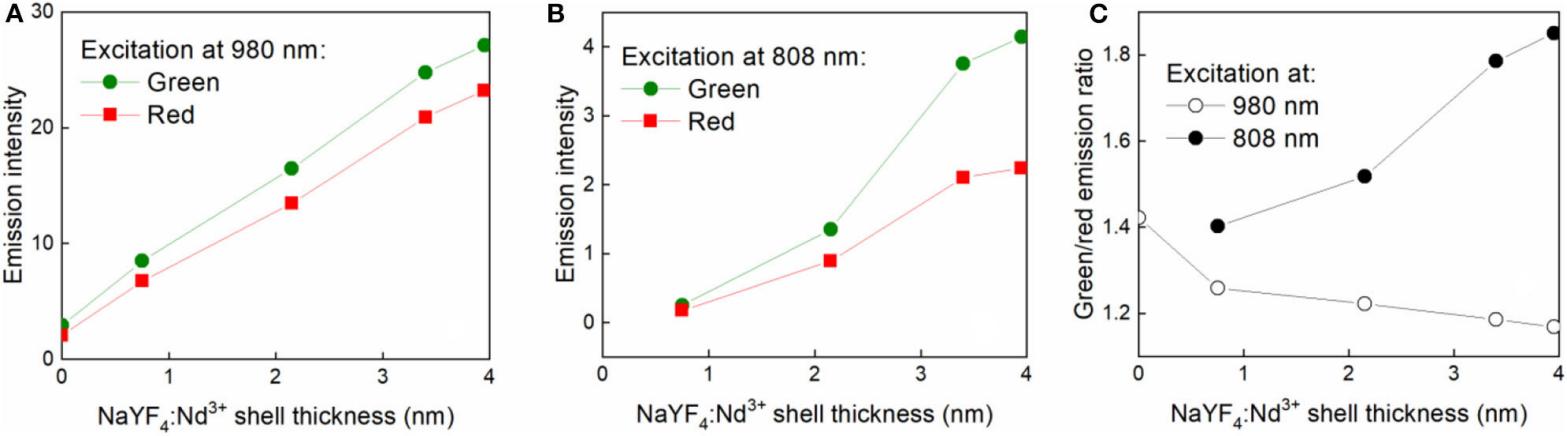
Dependence of green (541 nm) and red (655 nm) emission intensity of nanoparticles (1 mg/ml) in hexane/water emulsion stabilized with Igepal CO-520 on NaYF_4_:Nd^3+^ shell thickness at **(A)** 980 and **(B)** 808 nm excitation. **(C)** Gresen-to-red emission intensity ratio for different excitation wavelengths measured for different shell thickness; average power density of both lasers ~30 W/cm^2^.

**Figure 6:**
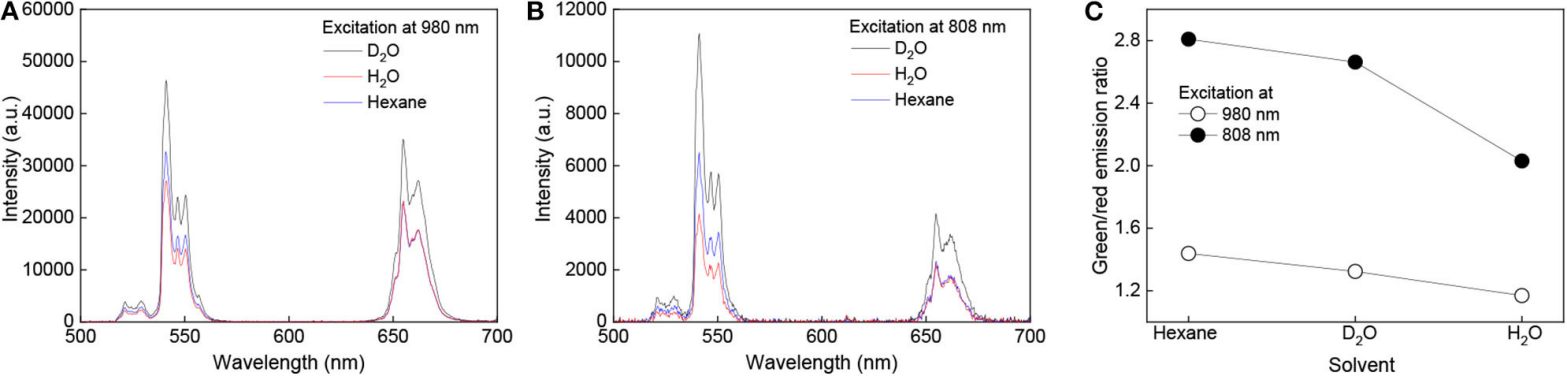
Photoluminescence upconversion spectra of NaYF_4_:Yb^3+^/Er^3+^@NaYF_4_:Nd^3+^(0.7 mmol) core-shell nanoparticles (1 mg/ml) in D_2_O (black), water (red), and hexane (blue) at **(A)** 808 and **(B)** 980 nm excitation. **(C)** Green-to-red emission intensity ratio for different solvents; average power density of both lasers ~30 W/cm^2^.

The emission spectra acquired at 980 nm excitation wavelength showed typical emission lines of Er^3+^ at 520 nm (^2^*H*_11/2_ → ^2^*I*_15/2_), 541 nm (^4^*S*_3/2_ → ^2^*I*_15/2_), and 655 nm (^4^*F*_9/2_ → ^2^*I*_15/2_) (Figure 4). After growth of the NaYF_4_:Nd^3+^ shell around the NaYF_4_:Yb^3+^/Er^3+^ core, the emission intensity of green and red light of NaYF_4_:Yb^3+^/Er^3+^@NaYF_4_:Nd^3+^(0.1 mmol), NaYF_4_: Yb^3+^/Er^3+^@NaYF_4_:Nd^3+^(0.3 mmol), NaYF_4_:Yb^3+^/Er^3+^@NaYF_4_:Nd^3+^(0.5 mmol) and NaYF_4_: Yb^3+^/Er^3+^@NaYF_4_:Nd^3+^(0.7 mmol) increased by 2.9×, 5.7×, 8.6×, and 9.4× and by 3.3×, 6.6×, 10.5×, and 11.9×, respectively, compared to that of the starting NaYF_4_:Yb^3+^/Er^3+^ cores (Figure 4A). This increase was linear for both green and red light (Figure 5A). It is worth of mentioning that this dependence can differ for various Yb/Er ratios and UCNP sizes, as shown in our previous paper (Podhorodecki et al., 2018). Under 808 nm excitation, characteristic peaks of green and red light originating from Er^3+^ ions were observed in the spectrum of the NaYF_4_:Yb^3+^/Er^3+^@NaYF_4_:Nd^3+^(0.1 mmol) nanoparticles. With increasing shell thickness in the NaYF_4_:Yb^3+^/Er^3+^@NaYF_4_:Nd^3+^(0.3 mmol), NaYF_4_:Yb^3+^/Er^3+^@NaYF_4_:Nd^3+^(0.5 mmol), and NaYF_4_:Yb^3+^/Er^3+^@NaYF_4_:Nd^3+^(0.7 mmol) particles, the intensity of green and red emission increased by 5.5×, 15.3×, and 16.9× and by 5.1×, 12.1×, and 12.9×, respectively, compared to that of the NaYF_4_:Yb^3+^/Er^3+^@NaYF_4_:Nd^3+^(0.1 mmol) particles (Figure 4B). The largest intensity increase in green and red emission was observed in the NaYF_4_:Yb^3+^/Er^3+^@NaYF_4_:Nd^3+^(0.5 mmol) particles, while it was smallest in the NaYF_4_:Yb^3+^/Er^3+^@NaYF_4_:Nd^3+^(0.7 mmol) particles (Figure 5B). This result can be explained by small diffusion efficiency of Nd^3+^ ions at the core-shell interface and small number of Nd^3+^ ions at low concentration in the shell. This reduces efficient energy transfer from Nd^3+^ to Er^3+^ ions located in UCNP core. Increasing Nd^3+^ concentration increases the probability of non-radiative energy transfer as the intermixing becomes more efficient and number Nd^3+^ ions increases as well. Green/red emission intensity ratio was significantly different at different excitation wavelengths (Figures 5C, 6C). The 808 nm excitation wavelength favored green luminescence. As expected, effect was stronger with increasing shell thickness, due to higher Nd^3+^ content in UCNPs. Let us note that intensity of the photoluminescence emission at 808 nm excitation was lower compared to 980 nm excitation (Figure 4). This difference can be attributed to different amount of Nd^3+^ and Yb^3+^ ions in the core-shell structure of the NaYF_4_:Yb^3+^/Er^3+^@NaYF_4_:Nd^3+^ nanoparticles. The Yb^3+^ ions that absorb light at 980 nm were present in the core, while the Nd^3+^ ions, absorbing photons at 808 nm and transferring the absorbed energy to Yb^3+^, were localized in the shell. The NaYF_4_:Nd^3+^ shell, which protects the particle surface from quenchers, facilitates enhancement of upconversion photoluminescence at 980 nm; at the same time, the presence of Nd^3+^ ions in the shell allows excitation at 808 nm, though these ions remain vulnerable to the surface quenchers. This, and additional energy losses during energy transfer between Nd^3+^ and Yb^3+^ ions, can explain the observed lower photoluminescence emission intensity at 808 nm compared to 980 nm excitation.

The upconversion photoluminescence spectra of the NaYF_4_:Yb^3+^/Er^3+^@NaYF_4_:Nd^3+^(0.7 mmol) particles (1 mg/ml) were measured in hexane, hexane/water, and hexane/D_2_O emulsions at 808 and 980 nm excitation and power density ~30 W/cm^2^. Significant enhancement of the luminescence intensity was observed in hexane and hexane/D_2_O compared to that in hexane/water emulsion. The green luminescence intensity of NaYF_4_:Yb^3+^/Er^3+^@NaYF_4_:Nd^3+^ (0.7 mmol) in hexane/D_2_O emulsion and hexane at 808 nm excitation was ~2.7- and 1.6-fold higher, respectively, than that of particles dispersed in hexane/water emulsion (Figure 6A). This enhancement can be explained by substitution of the O-H luminescence quencher with O-D. The energy of the O-D stretching vibration (2,500 cm^−1^) is lower than that of O-H (3,450 cm^−1^), which leads to a decrease in multiphoton relaxation rate in the case of D_2_O and enhancement of luminescence (Luwang et al., 2010). The same mechanism could also explain the high luminescence intensity of the NaYF_4_:Yb^3+^/Er^3+^@NaYF_4_:Nd^3+^ (0.7 mmol) particles in hexane. Additionally, in this case, the energy of the C-H stretching vibration (2,900 cm^−1^) is lower than that of O-H and thus more phonons are needed in hexane to dissipate the excitation energy and depopulate excited carriers from green to red energy level (Figure 6C). This makes green emission more intense. The effect is stronger under excitation at 808 nm due to the proximity of the luminescence sensitizer (Nd^3+^) and solvent molecules. In the case of 980 nm excitation, the green luminescence intensity was 7× and 1.2× higher in the hexane/D_2_O emulsion and hexane, respectively, due to the small effect of hexane on the Yb^3+^ ions (Figure 6B).

### UCNP Upconversion Spectra With a Pulsed Excitation

Upconversion luminescence of the NaYF_4_:Yb^3+^/Er^3+^@NaYF_4_:Nd^3+^, NaYF_4_: Yb^3+^/Er^3+^@NaYF_4_:Nd^3+^-PEG_5,475_, and NaYF_4_: Yb^3+^/Er^3+^@NaYF_4_:Nd^3+^-PEG_5,475_-RGDS nanoparticles was confirmed by the emission spectra at 980 nm obtained with excitation by 140 fs pulses (Figure S6). The particles exhibited two typical upconversion peaks at 535 and 635 nm (Kostiv et al., 2015).

### Cell Monitoring

In this set of experiments, Hep-G2 cells were incubated with all types of nanoparticles to monitor their cell distribution by confocal microscopy. Various volumes of the particle dispersions (20, 40, 60, 100, and 200 μl) were added to the DMEM-based medium containing the cells. A volume amounting to 200 μl was found to be optimal, avoiding damage to cells and at the same time providing good particle visibility; this volume was therefore used in further biological experiments.

**Figure 7:**
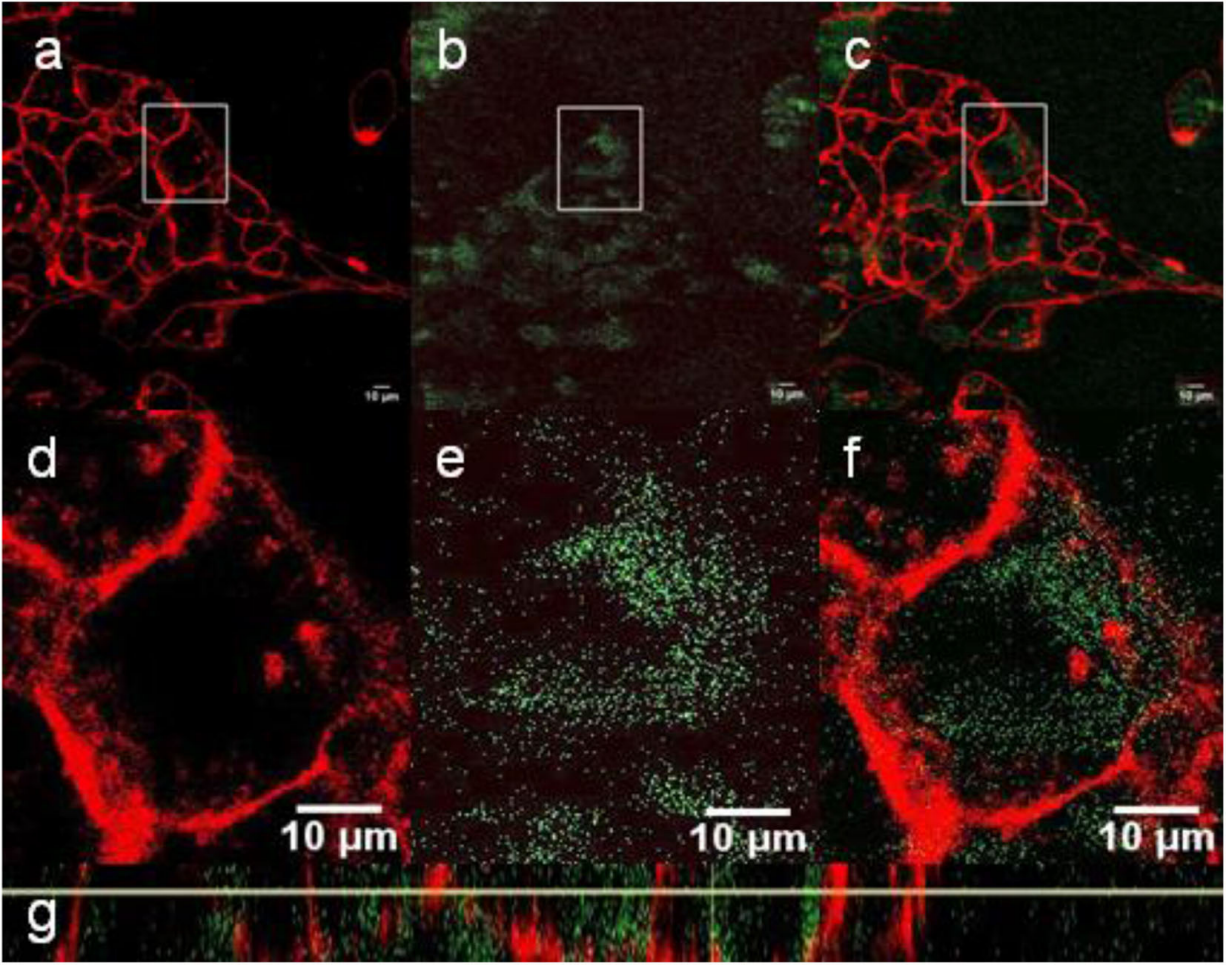
**(a–f)** Confocal micrographs showing the distribution of NaYF_4_:Yb^3+^/Er^3+^@NaYF_4_:Nd^3+^ nanoparticles (200 μl) in Hep-G2 cells at 980 nm excitation with a laser power of 30–50 mW. **(d–f)** Detailed micrographs of **(a–c)**. **(a,d)** CellMask™ deep red cell-stained cell membrane, **(b,e)** nanoparticles (green), and **(c,f)** overlay of **(a,b,d,e)**. **(g)** The xz-plane of NaYF_4_:Yb^3+^/Er^3+^@NaYF_4_:Nd^3+^ nanoparticle-labeled Hep-G2 cells.

Upconversion luminescence was determined after incubation of the NaYF_4_:Yb^3+^/Er^3+^@NaYF_4_:Nd^3+^, NaYF_4_:Yb^3+^/Er^3+^@NaYF_4_:Nd^3+^-PEG_5,475_, and NaYF_4_:Yb^3+^/Er^3+^@NaYF_4_:Nd^3+^-PEG_5,475_-RGD particles with Hep-G2 cells, the membranes of which were stained with CellMask™ deep red. Localization of the particles in the cells was demonstrated within the confocal plane by confocal microscopy in the range of 500-670 nm using z-scan via the xy and xz plane, as exemplified on the NaYF_4_:Yb^3+^/Er^3+^@NaYF_4_:Nd^3+^ particles (Figure 7). This approach allowed us to determine the biodistribution of the particles, particularly on the cell membrane and in the cell cytosol. Since the emission spectra of nanoparticles in the cells at 535 and 640 nm under 980 nm pulsed excitation with 140 fs pulses corresponded to those of neat particles, the presence of NaYF_4_:Yb^3+^/Er^3+^@NaYF_4_:Nd^3+^ particles in the Hep-G2 cells was proven (Figure S6). Moreover, penetration of the NaYF_4_:Yb^3+^/Er^3+^@NaYF_4_:Nd^3+^ nanoparticles into the cells was confirmed by particle localization inside the cells, which were contoured in their plasma membranes by CellMask™ deep red. Artificial nanoparticle adherence to the top of cell surface is excluded by colocalization of the two confocal images of cell section (nanoparticle emission vs. CellMask™ deep red emission) within the xz plane (Figure 7g).

**Figure 8:**
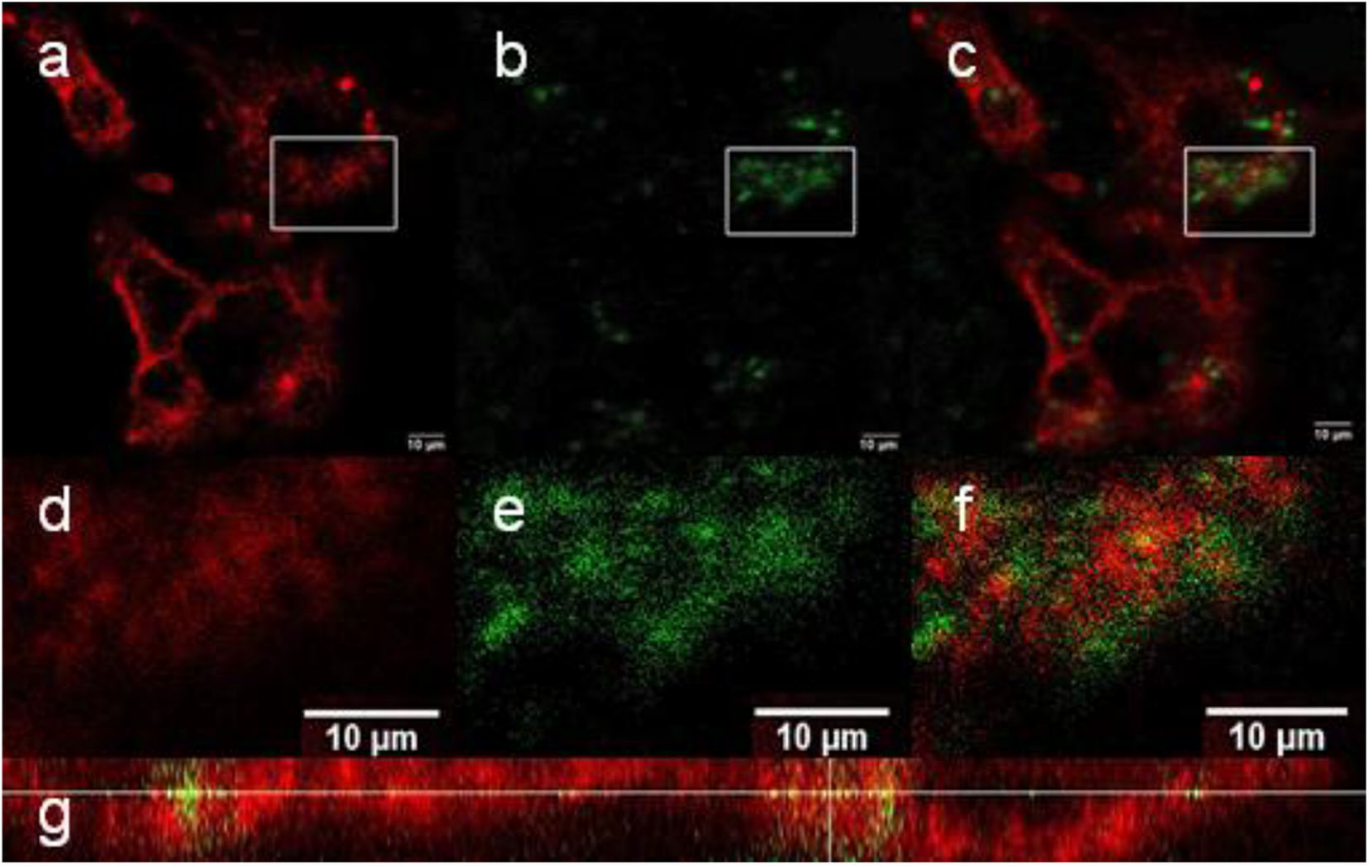
**(a–f)** Confocal micrographs showing the distribution of NaYF_4_:Yb^3+^/Er^3+^@NaYF_4_:Nd^3+^-PEG_5,475_-RGDS nanoparticles in Hep-G2 cells at 980 nm excitation with a laser power of 30–50 mW. **(d–f)** Detailed micrographs of **(a–c)**. **(a,d)** CellMask™ deep red cell-stained cell membrane, **(b,e)** nanoparticles, and **(c,f)** overlay of **(a,b,d,e)**. **(g)** The xz-plane of NaYF_4_:Yb^3+^/Er^3+^@NaYF_4_:Nd^3+^-PEG_5,475_-RGDS nanoparticle-labeled Hep-G2 cells.

**Figure 9:**
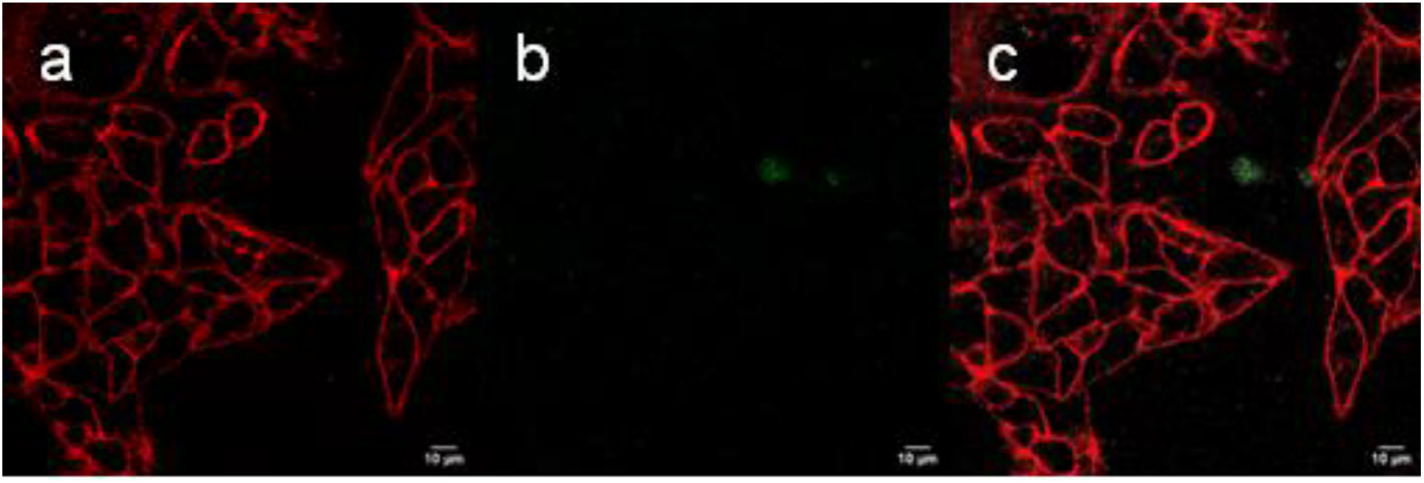
**(a–c)** Confocal micrographs showing the distribution of NaYF_4_:Yb^3+^/Er^3+^@NaYF_4_:Nd^3+^-PEG_5,475_-Alk nanoparticles in Hep-G2 cells at 980 nm excitation with a laser power of 30–50 mW. **(a)** CellMask™ deep red cell-stained cell membrane, **(b)** nanoparticles, and **(c)** overlay of **(a,b)**.

In contrast to the NaYF_4_:Yb^3+^/Er^3+^@NaYF_4_:Nd^3+^ nanoparticles, the NaYF_4_:Yb^3+^/Er^3+^@PEG_5,475_-RGDS nanoparticles adhered to the cell membrane (Figure 8), while the NaYF_4_:Yb^3+^/Er^3+^@NaYF_4_:Nd^3+^-PEG_5,475_-Alk particles were located outside of the cells (Figure 9). Colocalization of the NaYF_4_:Yb^3+^/Er^3+^@PEG_5,475_-RGDS particles with CellMask™ deep red-marked cell membranes was apparent only within the xz plane (Figure 8g). Hence, unlike to the NaYF_4_:Yb^3+^/Er^3+^@NaYF_4_:Nd^3+^ nanoparticles, the NaYF_4_:Yb^3+^/Er^3+^@PEG_5,475_-RGDS particles were absent inside the cells in the xy images, but colocalizations occurred at the edge of the cells within the xz plane. Finally, colocalizations of NaYF_4_:Yb^3+^/Er^3+^@NaYF_4_:Nd^3+^-PEG_5,475_-Alk nanoparticles were not observed, either intracellularly or within the plasma membrane (Figure 9). Both z- and λ-scans (emission spectrum) confirmed that the emission originated from particle upconversion, distinguishing the emission from background cell autofluorescence, as exemplified by the NaYF_4_:Yb^3+^/Er^3+^@NaYF_4_:Nd^3+^ particles (Figure S6).

As a result, UCNP surface engineering with PEG and/or RGDS peptide enabled us to target the particles in various cell compartments that are important for prospective biomedical applications, in particular photodynamic therapy of tumors.

## Conclusions

In conclusion, the hexagonal β-NaYF_4_ phase of NaYF_4_:Yb^3+^/Er^3+^@NaYF_4_:Nd^3+^ core-shell nanoparticles was successfully synthesized by thermal coprecipitation of lanthanide precursors. The number-average size of the core particles was ~24 nm and the shell thickness could be adjusted in the range of 1–4 nm, depending on the amount of precursors used in the synthesis. The particles were monodisperse, which is important in terms of controlling their properties, reproducible photoluminescence, and improved performance in drug release systems. To make both the starting NaYF_4_:Yb^3+^/Er^3+^ core and NaYF_4_:Yb^3+^/Er^3+^@NaYF_4_:Nd^3+^ core-shell nanoparticles long-term dispersible in PBS, they were coated with in-house synthesized Ner-PEG-Alk. While bisphosphonate terminal groups of neridronate strongly conjugated to the surface of UCNPs, alkyne groups were available for the copper-catalyzed click reaction with azido-RGDS peptide. The presence of PEG on the UCNPs was confirmed by FTIR analysis and TGA, which proved that the NaYF_4_:Yb^3+^/Er^3+^@NaYF_4_:Nd^3+^-PEG_5,475_-Alk nanoparticles contained up to 25 wt.% PEG. Spectroscopic studies showed significant difference in the activator luminescence (Er^3+^) upon the excitation of the first (Yb^3+^) and the second (Nd^3+^) sensitizer. Interestingly, in the latter, the luminescence spectra strongly depended on the shell thickness. Precise control of this parameter could be potentially used for tuning the optical properties of NaYF_4_:Yb^3+^/Er^3+^@NaYF_4_:Nd^3+^ nanoparticles. We have also observed a noticeable NaYF_4_:Yb^3+^/Er^3+^@NaYF_4_:Nd^3+^ luminescence quenching in water, predominantly for the green luminescence bands.

The particles were non-cytotoxic as confirmed by the trypan blue exclusion test. It was interesting to note that the NaYF_4_:Yb^3+^/Er^3+^@NaYF_4_:Nd^3+^ particles were well-internalized by the Hep-G2 cells, while the NaYF_4_:Yb^3+^/Er^3+^@NaYF_4_:Nd^3+^-PEG_5,475_-RGDS nanoparticles accumulated on the cell membranes, which is in contrast to the NaYF_4_:Yb^3+^/Er^3+^@NaYF_4_:Nd^3+^-PEG_5,475_-Alk particles that were neither engulfed by the cells nor tightly adsorbed on the cell membrane surface. This finding confirmed that the RGDS peptide was effective for adhesion of UCNPs to the cell membranes. Specific binding of the NaYF_4_:Yb^3+^/Er^3+^@NaYF_4_:Nd^3+^-PEG_5,475_-RGDS nanoparticles to membranes of various cell types can also be achieved using antibodies, which might be important for addressing specific cell types. In contrast, the NaYF_4_:Yb^3+^/Er^3+^@NaYF_4_:Nd^3+^ particles (without RGDS) could be useful for example for photodynamic therapy (PDT) of tumors, if a proper photosensitizer is attached to the particle coating. PDT with excitation at 808 nm is a newly developing field, as 808 nm light can penetrate tissue up to a depth of 5 cm. Such an application would greatly increase the number of conditions that can be treated using infrared PDT. Moreover, NaYF_4_:Yb^3+^/Er^3+^@NaYF_4_:Nd^3+^-based systems can be prospectively modified to serve as drug delivery vehicles for any general therapy, releasing the attached or adsorbed drug in the affected cells and/or tissues.

## Data Availability Statement

All datasets generated for this study are included in the article/Supplementary Material.

## Author Contributions

UK synthesized and characterized the particles. HE cultivated and monitored the cells. BK measured the optical spectra. MŠ investigated morphology and crystal structure of the particles. VP synthesized RGDS peptide. AP measured upconversion photoluminescence spectra. PJ measured upconversion spectra with a pulsed excitation. DH supervised the work and together with UK wrote the publication.

## Conflict of Interest

The authors declare that the research was conducted in the absence of any commercial or financial relationships that could be construed as a potential conflict of interest.
