# Supplementary material for: Monodisperse Core-Shell NaYF4:Yb3+/Er3+@NaYF4:Nd3+-PEG-GGGRGDSGGGY-NH2 Nanoparticles Excitable at 808 and 980 nm: Design, Surface Engineering, and Application in Life Sciences
Source: Front Chem. 2020 Jun 12;8:497. doi: 10.3389/fchem.2020.00497 (PMC7303004; doi:10.3389/fchem.2020.00497)
Supplement: Supplementary file 1 [file Data_Sheet_1.PDF]

SUPLEMENTARY MATERIAL

Table S1. Number-average diameter (D_n), uniformity (dispersity *Đ*) of core and core-shell nanoparticles and $NaYF_4:Nd^{3+}$ shell thickness.

Nanoparticles	D_n (nm)	*Đ*	Shell thickness (nm)
$NaYF_4:Yb^{3+}/Er^{3+}$	24.2±0.9	1.00	-
$NaYF_4:Yb^{3+}/Er^{3+}$@ $NaYF_4:Nd^{3+}$(0.1 mmol)	25.7±1.3	1.01	0.75
$NaYF_4:Yb^{3+}/Er^{3+}$@ $NaYF_4:Nd^{3+}$(0.3 mmol)	28.5±1.8	1.01	2.15
$NaYF_4:Yb^{3+}/Er^{3+}$@ $NaYF_4:Nd^{3+}$(0.5 mmol)	31.0±1.4	1.01	3.40
$NaYF_4:Yb^{3+}/Er^{3+}$@ $NaYF_4:Nd^{3+}$(0.7 mmol)	32.1±1.8	1.01	3.95

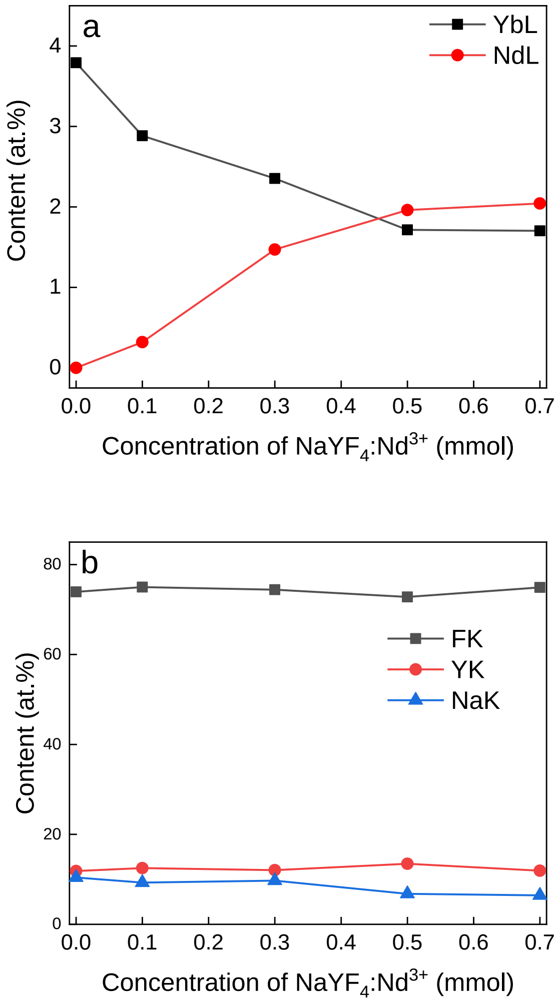

Figure S1. Dependence of (a) Yb, Nd and (b) F, Y, Na content in the $NaYF_4:Yb^{3+}/Er^{3+}$@$NaYF_4:Nd^{3+}$ nanoparticles on concentration of $NaYF_4:Nd^{3+}$; composition was determined by TEM/EDX.

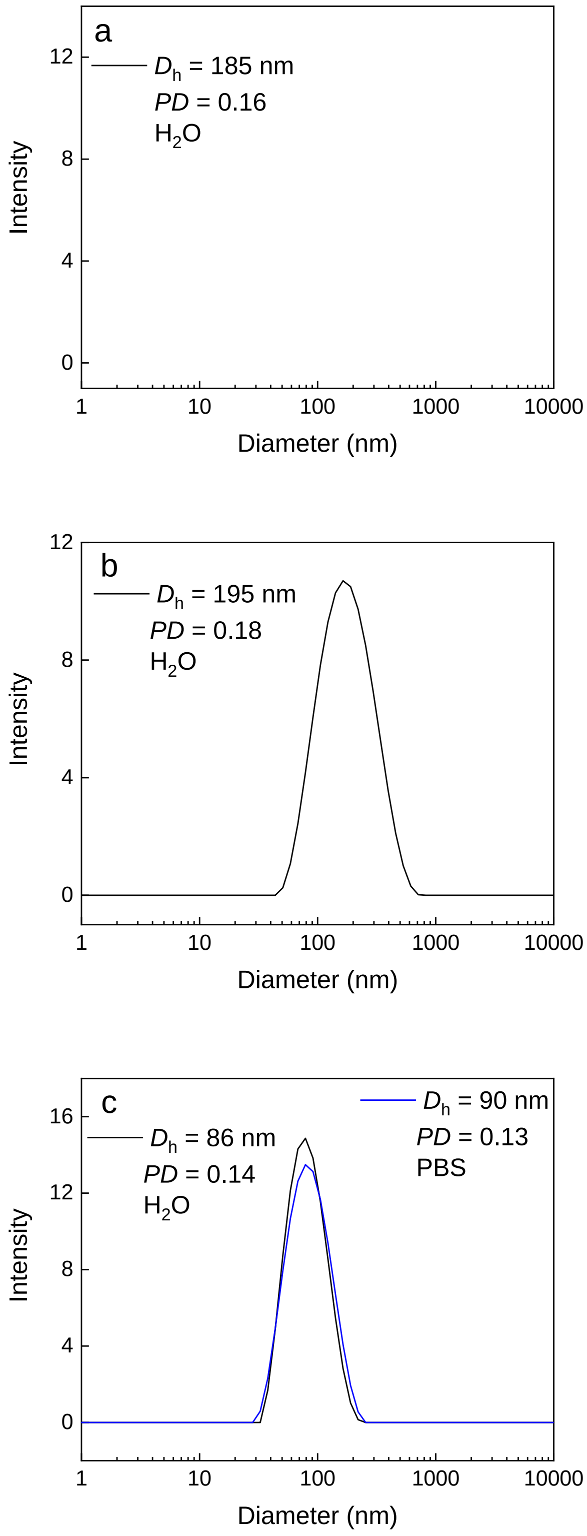

a
D_h = 185 nm
PD = 0.16
H_2O
Intensity
0
4
8
12
1
10
100
1000
10000
Diameter (nm)
b
D_h = 195 nm
PD = 0.18
H_2O
Intensity
0
4
8
12
1
10
100
1000
10000
Diameter (nm)
c
D_h = 86 nm
PD = 0.14
H_2O
D_h = 90 nm
PD = 0.13
PBS
Intensity
0
4
8
12
16
1
10
100
1000
10000
Diameter (nm)

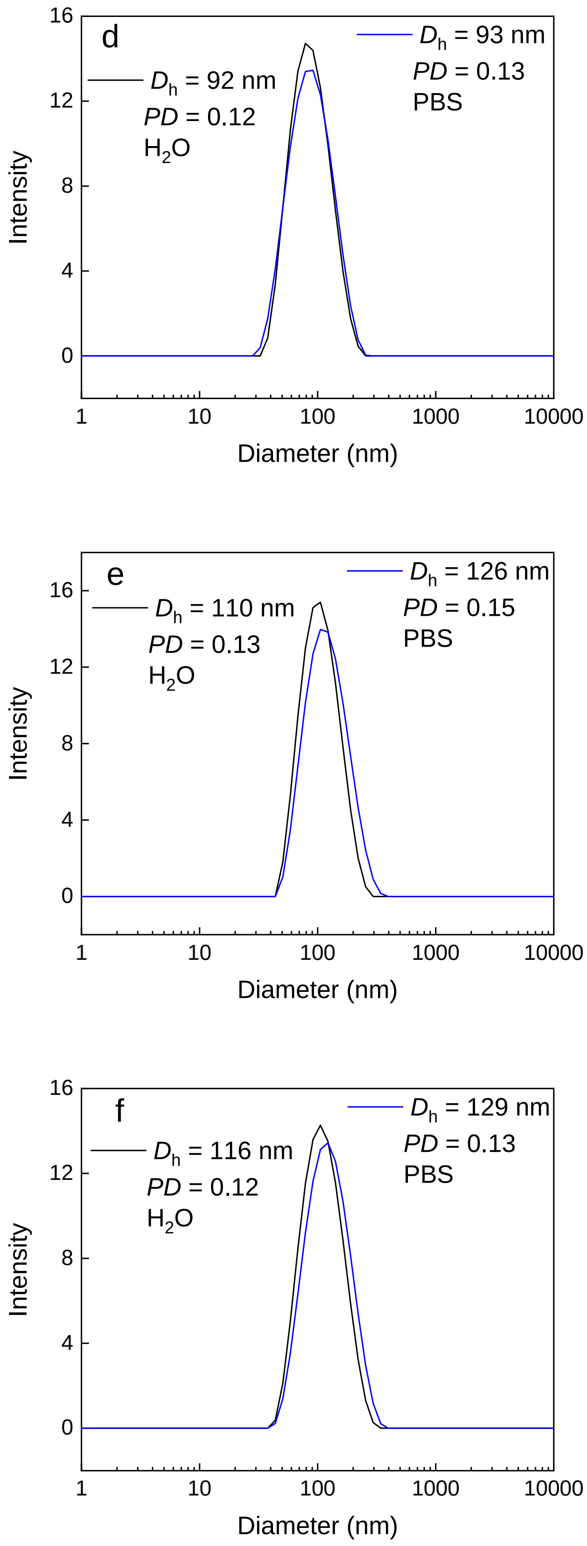
d
D_h = 92 nm
PD = 0.12
H_2O
D_h = 93 nm
PD = 0.13
PBS
e
D_h = 110 nm
PD = 0.13
H_2O
D_h = 126 nm
PD = 0.15
PBS
f
D_h = 116 nm
PD = 0.12
H_2O
D_h = 129 nm
PD = 0.13
PBS
Intensity
Diameter (nm)
16
12
8
4
0
1
10
100
1000
10000

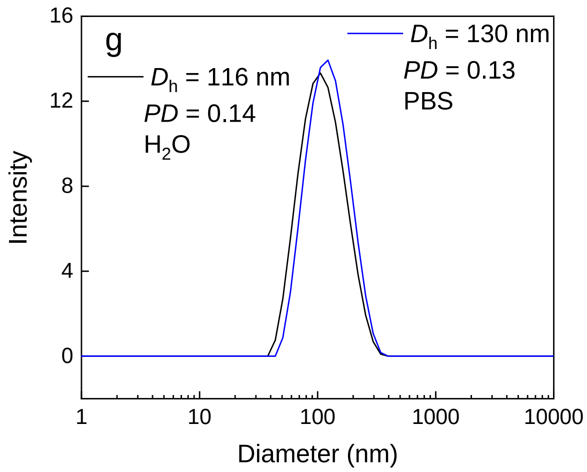

Figure S2. Size distribution of (a) $NaYF_4:Yb^{3+}/Er^{3+}$, (b) $NaYF_4:Yb^{3+}/Er^{3+}$@$NaYF_4:Nd^{3+}$, (c) $NaYF_4:Yb^{3+}/Er^{3+}$-$PEG_{3,815}$-Alk, (d) $NaYF_4:Yb^{3+}/Er^{3+}$-$PEG_{5,475}$-Alk, (e) $NaYF_4:Yb^{3+}/Er^{3+}$@$NaYF_4:Nd^{3+}$-$PEG_{3,815}$-Alk, (f) $NaYF_4:Yb^{3+}/Er^{3+}$@$NaYF_4:Nd^{3+}$-$PEG_{5,475}$-Alk, and (g) $NaYF_4:Yb^{3+}/Er^{3+}$@$NaYF_4:Nd^{3+}$-$PEG_{5,475}$-RGD nanoparticles in water (black) and 0.01 M PBS (blue); calculated from intensity-weighted distribution function.

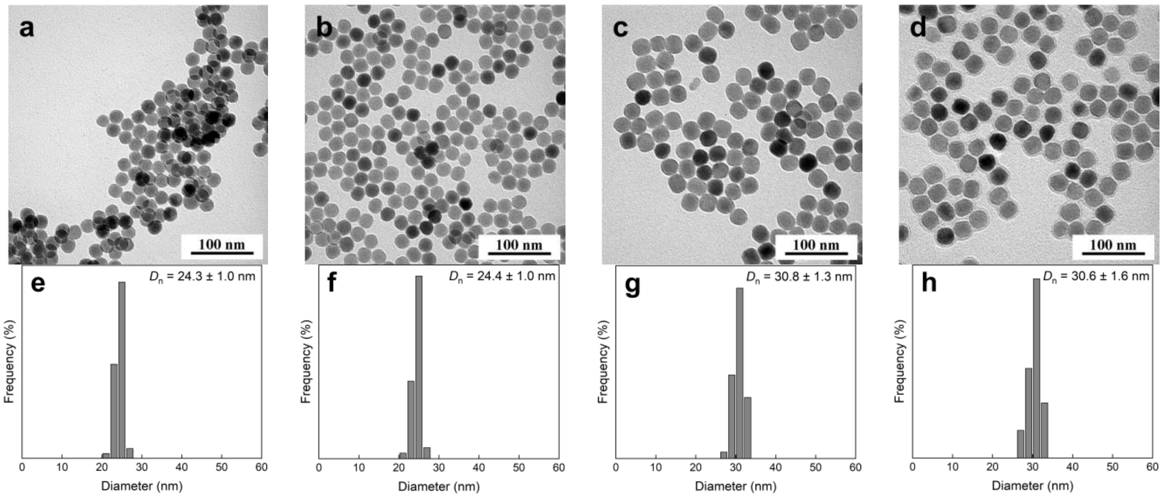

Figure S3. (a-d) TEM/BF micrographs and (e-h) particle size distributions of (a, e) $NaYF_4:Yb^{3+}/Er^{3+}$, (b, f) $NaYF_4:Yb^{3+}/Er^{3+}$-$PEG_{5,475}$-Alk, (c, g) $NaYF_4:Yb^{3+}/Er^{3+}$@$NaYF_4:Nd^{3+}$(0.5 mmol), and (d, h) $NaYF_4:Yb^{3+}/Er^{3+}$@$NaYF_4:Nd^{3+}$(0.5 mmol)-$PEG_{5,475}$-Alk particles. D_n is shown in the upper right corners of the histograms.

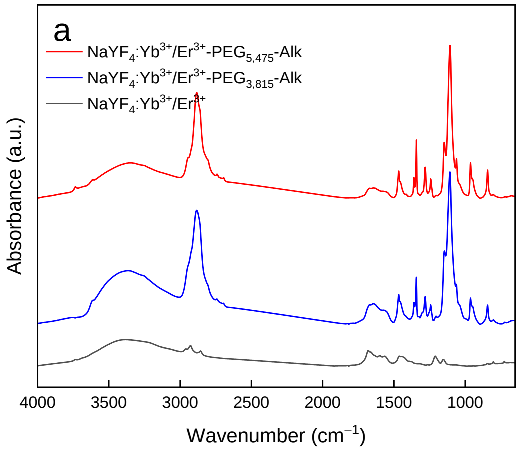
a
NaYF4:Yb3+/Er3+-PEG5,475-Alk
NaYF4:Yb3+/Er3+-PEG3,815-Alk
NaYF4:Yb3+/Er3+
Absorbance (a.u.)
4000 3500 3000 2500 2000 1500 1000
Wavenumber (cm−1)

a

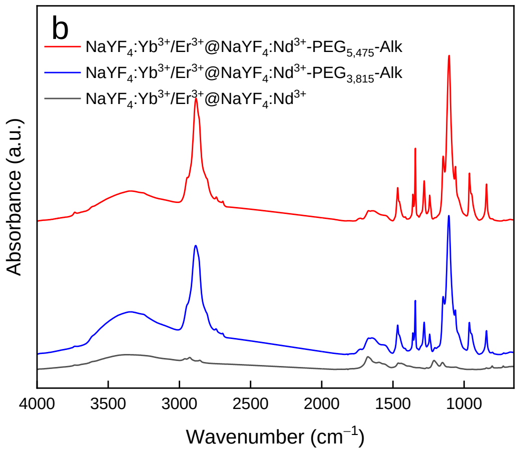
b
NaYF4:Yb3+/Er3+@NaYF4:Nd3+-PEG5,475-Alk
NaYF4:Yb3+/Er3+@NaYF4:Nd3+-PEG3,815-Alk
NaYF4:Yb3+/Er3+@NaYF4:Nd3+
Absorbance (a.u.)
4000 3500 3000 2500 2000 1500 1000
Wavenumber (cm−1)

b

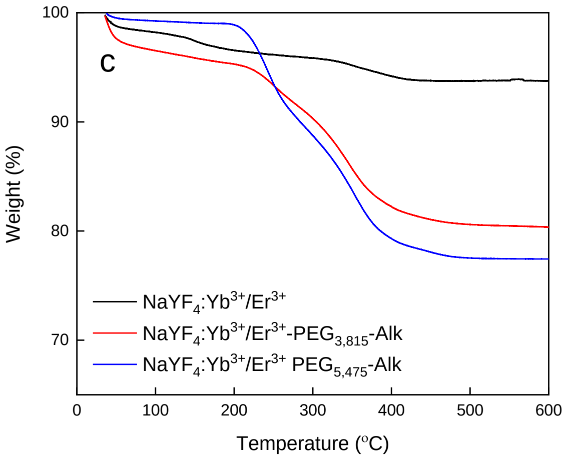
c
100
90
80
70
Weight (%)
NaYF4:Yb3+/Er3+
NaYF4:Yb3+/Er3+-PEG3,815-Alk
NaYF4:Yb3+/Er3+ PEG5,475-Alk
0 100 200 300 400 500 600
Temperature (oC)

c

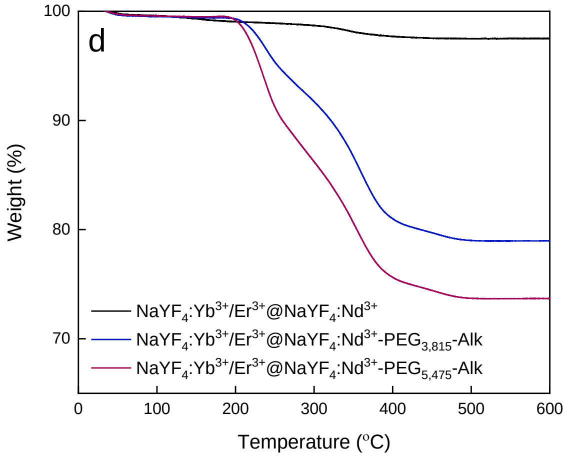

d

Figure S4. (a, b) ATR FTIR and (c, d) TGA spectra of unmodified and PEG-coated (a, c) $NaYF_4$:Yb^{3+}/Er^{3+} core and (b, d) $NaYF_4$:Yb^{3+}/Er^{3+}@$NaYF_4$:Nd^{3+} core-shell nanoparticles.

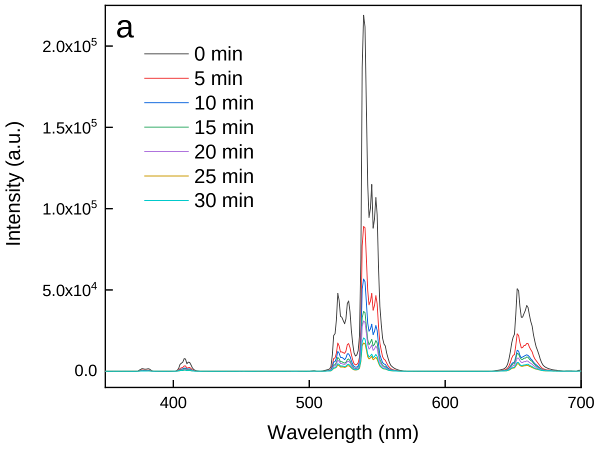

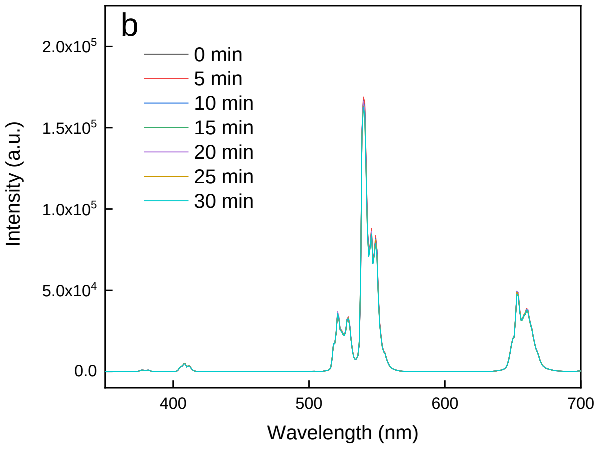

Figure S5. Photoluminescent upconversion spectra of $NaYF_4$:Yb^{3+}/Er^{3+}@$NaYF_4$:Nd^{3+}(0.5 mmol) core-shell nanoparticles (1 mg/ml) in (a) hexane and (b) hexane/water emulsion stabilized with Igepal CO-520; excitation at 980 nm and power density 0.5 W/cm^2.

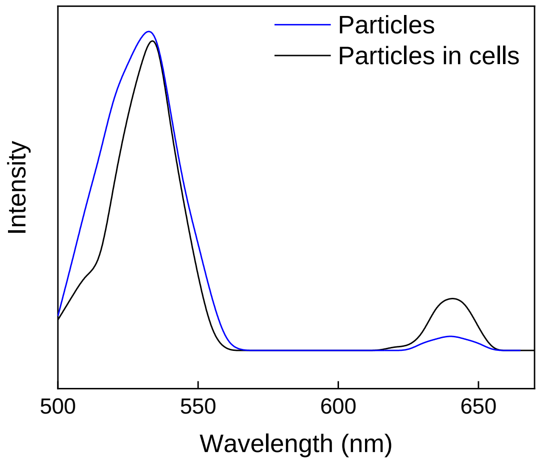

Figure S6. Emission spectra of $NaYF_4$:Yb^{3+}/Er^{3+}@$NaYF_4$:Nd^{3+} nanoparticles (blue) and nanoparticles in Hep-G2 cells (black) determined by confocal microscopy at 980 nm excitation with pulse width ~140 fs.
